# Supplementary material for: Do the associations of body mass index and waist circumference with back pain change as people age? 32 years of follow-up in a British birth cohort
Source: BMJ Open. 2020 Dec 12;10(12):e039197. doi: 10.1136/bmjopen-2020-039197 (PMC7735102; doi:10.1136/bmjopen-2020-039197)
Supplement: Supplementary data [file bmjopen-2020-039197supp001.pdf]

# Supplementary information: Do the associations of body mass index and waist circumference with back pain change as people age? 32 years of follow-up in a British birth cohort

Stella G Muthuri, Rachel Cooper, Diana Kuh & Rebecca Hardy

**Supplementary methods:**

**Questions used to assess back pain in the MRC National Survey of Health and Development**

***Ages 36 and 43 years [nurse interviews]:***

Do you have any of the following (sciatica, lumbago or recurring backache) all or most of the time?

0 No

1 Yes

***Ages 53 and 60-64 years [nurse interviews]:***

In the last 12 months, have you had sciatica, lumbago or severe backache?

0 No

1 Yes

***Age 68 years [self-completion questionnaire]:***

In the *last month,* have you had any ache or pain which has lasted for *one day or longer*? (Please do not include pain occurring only during the course of a feverish illness such as flu)

0 No

1 Yes

Below you will find four diagrams of the body.

Please **shade** in all the places where you have felt or feel the aches and pains.

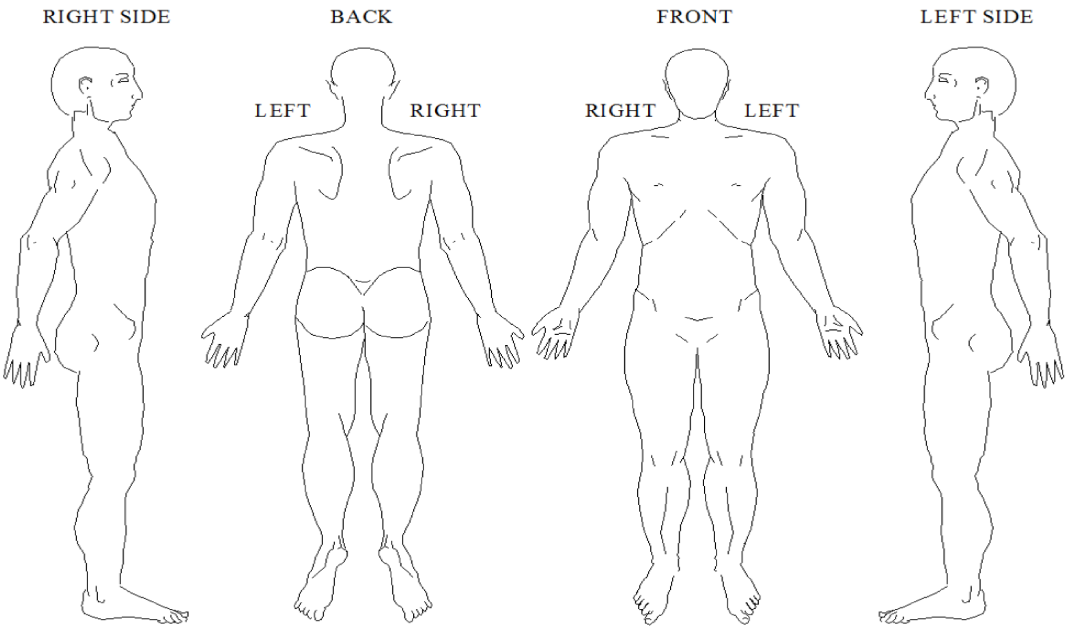




Muthuri S, *et al. BMJ Open* 2020; 10:e039197. doi: 10.1136/bmjopen-2020-039197

**Table S1: Number of MRC National Survey of Health and Development participants contributing data at each age and number with missing data[a]**

| Age | Total N contributing data at specified age (no. of males; no. of females) | N with data on back pain but missing data on BMI/WC at specified age | N with data on back pain and BMI/WC at specified age but missing data on covariates |
|---|---|---|---|
| 36y | 3093 (1540; 1553) | 48 | 175 |
| 43y | 2940 (1483; 1457) | 52 | 259 |
| 53y | 2700 (1332; 1368) | 44 | 243 |
| 60-64y | 1816 (872; 944) | 29 | 390 |
| 68-69y | 1777 (860; 917) | 485 | 158 |

[a] A total of 3426 MRC NSHD participants were included in the main analyses. Participant's data could be included at a specified age if there was complete information on back pain, BMI, waist circumference and all covariates at that age
BMI: Body mass index; WC: waist circumference



Muthuri S, *et al. BMJ Open* 2020; 10:e039197. doi: 10.1136/bmjopen-2020-039197

**Table S2: Characteristics of the MRC National Survey of Health and Development sample included in analysis [a]**

| | Male | Female |
|---|---|---|
| **Highest education level achieved by age 26y, N (%)** | | |
| A-level/equivalent or above | 706 (41.0) | 463 (27.2) |
| O-level/equivalent or below | 357 (20.7) | 587 (34.5) |
| None | 660 (38.3) | 653 (38.3) |
| **Own occupational class at age 53y, N (%)** | | |
| High (I/II) | 853 (49.5) | 593 (34.8) |
| Middle (IIINM/IIIM) | 662 (38.4) | 740 (43.5) |
| Low (IV/V) | 208 (12.1) | 370 (21.7) |
| **Time varying covariates[b]** | | |
| **Height (cm), mean (SD), at age:** | | |
| 36y | 175.4 (6.5) | 162.4 (6.0) |
| 43y | 175.2 (6.6) | 162.4 (6.2) |
| 53y | 174.7 (6.5) | 161.6 (5.9) |
| 60-64y | 174.8 (6.5) | 161.9 (5.9) |
| 69y | 174.0 (6.4) | 160.7 (5.9) |
| **Smoking status, N (%), at age:** | | |
| 36y | | |
| Current | 530 (34.4) | 524 (33.7) |
| Ex | 617 (40.1) | 518 (33.4) |
| Never | 393 (25.5) | 511 (32.9) |
| 43y | | |
| Current | 460 (31.0) | 417 (28.6) |
| Ex | 522 (35.2) | 373 (25.6) |
| Never | 501 (33.8) | 667 (45.8) |
| 53y | | |
| Current | 323 (24.3) | 315 (23.0) |
| Ex | 544 (40.8) | 391 (28.6) |
| Never | 465 (34.9) | 662 (48.4) |
| 60-64y | | |
| Current | 97 (11.1) | 105 (11.1) |
| Ex | 410 (47.0) | 322 (34.1) |
| Never | 365 (41.9) | 517 (54.8) |
| 68-69y | | |
| Current | 76 (8.8) | 65 (7.1) |
| Ex | 411 (47.8) | 314 (34.2) |
| Never | 373 (43.4) | 538 (58.7) |
| **Leisure time physical activity, N (%), at age:** | | |
| 36y | | |
| Inactive | 475 (30.8) | 651 (41.9) |
| Less active | 407 (26.4) | 376 (24.2) |
| Most active | 658 (42.7) | 526 (33.9) |
| 43y | | |

| | | | |
|---|---|---|---|
| | Inactive | 714 (48.2) | 805 (55.3) |
| | Less active | 354 (23.9) | 338 (23.2) |
| | Most active | 415 (28.0) | 314 (21.6) |
| 53y | | | |
| | Inactive | 627 (47.1) | 689 (50.4) |
| | Less active | 252 (18.9) | 227 (16.6) |
| | Most active | 453 (34.0) | 452 (33.0) |
| 60-64y | | | |
| | Inactive | 557 (63.9) | 585 (62.0) |
| | Less active | 118 (13.5) | 142 (15.0) |
| | Most active | 197 (22.6) | 217 (23.0) |
| 68-69y | | | |
| | Inactive | 491 (57.1) | 520 (56.7) |
| | Less active | 101 (11.7) | 141 (15.4) |
| | Most active | 268 (31.2) | 256 (27.9) |
| **Symptoms of anxiety and depression, N(%), at age:** | | | |
| 36y | | | |
| | No | 1482 (96.2) | 1420 (91.4) |
| | Yes | 58 (3.8) | 133 (8.6) |
| 43y | | | |
| | No | 1350 (91.0) | 1233 (84.6) |
| | Yes | 133 (9.0) | 224 (15.4) |
| 53y | | | |
| | No | 1147 (86.1) | 1035 (75.7) |
| | Yes | 185 (13.9) | 333 (24.3) |
| 60-64y | | | |
| | No | 765 (87.7) | 739 (78.3) |
| | Yes | 107 (12.3) | 205 (21.7) |
| 69y | | | |
| | No | 781 (90.8) | 764 (83.3) |
| | Yes | 79 (9.2) | 153 (16.7) |

SD: Standard Deviation; y: years

[a] Maximum N=3426 (this includes participants with a valid measure of back pain, BMI, waist circumference and each covariate for at least one age)

[b] See number of participants with valid data at specified age in Supplementary Table S1



Muthuri S, *et al. BMJ Open* 2020; 10:e039197. doi: 10.1136/bmjopen-2020-039197

**Table S3: Coefficients from the multilevel models for standardised BMI and waist circumference with back pain across adulthood (N=3426)**

| | BMI | | | |
|---|---|---|---|---|
| | Model 1 | | Model 2[a] | |
| | Log-odds (SE) | P value | Log-odds (SE) | P value |
| BMI (per SD) | 0.119 (0.071) | 0.03 | 0.112 (0.056) | 0.05 |
| Sex (female) | 0.221 (0.072) | 0.002 | 0.500 (0.102) | <0.001 |
| Age 43 | 0.683 (0.073) | <0.001 | 0.674 (0.074) | <0.001 |
| Age 53 | 1.008 (0.074) | <0.001 | 0.993 (0.076) | <0.001 |
| Age 60-64 | 0.869 (0.083) | <0.001 | 0.895 (0.087) | <0.001 |
| Age 68 | 0.786 (0.084) | <0.001 | 0.876 (0.088) | <0.001 |
| Age 43*BMI | -0.019 (0.071) | 0.8 | -0.009 (0.071) | 0.9 |
| Age 53*BMI | 0.037 (0.072) | 0.5 | 0.045 (0.072) | 0.5 |
| Age 60-64*BMI | 0.148 (0.082) | 0.07 | 0.148 (0.081) | 0.07 |
| Age 68*BMI | -0.040 (0.084) | 0.6 | -0.046 (0.084) | 0.6 |

| | Waist circumference | | | |
|---|---|---|---|---|
| | Model 1 | | Model 2[a] | |
| | Log-odds (SE) | P value | Log-odds (SE) | P value |
| WC (per SD) | 0.160 (0.057) | 0.005 | 0.129 (0.057) | 0.02 |
| Sex (female) | 0.221 (0.071) | 0.002 | 0.442 (0.102) | <0.001 |
| Age 43 | 0.684 (0.073) | <0.001 | 0.674 (0.074) | <0.001 |
| Age 53 | 1.010 (0.074) | <0.001 | 0.990 (0.076) | <0.001 |
| Age 60-64 | 0.875 (0.083) | <0.001 | 0.896 (0.087) | <0.001 |
| Age 68 | 0.787 (0.084) | <0.001 | 0.867 (0.088) | <0.001 |
| Age 43*WC | -0.048 (0.072) | 0.5 | -0.049 (0.072) | 0.5 |
| Age 53*WC | -0.011 (0.073) | 0.9 | -0.009 (0.073) | 0.9 |
| Age 60-64*WC | 0.076 (0.083) | 0.4 | 0.063 (0.083) | 0.5 |
| Age 68*WC | -0.050 (0.084) | 0.6 | -0.058 (0.084) | 0.5 |

*Note.* BMI: body mass index; WC: waist circumference; SE: standard error; SD: Standard Deviation

As BMI and WC are standardised and so have a mean of 0 and SD of 1, the main effects of age represent the estimate at the mean BMI

[a] Model 2: model 1 + education, occupational class and time-varying covariates (height, cigarette smoking status, physical activity and symptoms of anxiety and depression)

**Table S4: Associations between body composition measures[a] at age 60-64 and back pain at age 68 (n=1186)**

| | Model 1[b] | | Model 2[c] | |
|---|---|---|---|---|
| Sex-standardised body composition measures | Odds ratio (95%CI) | P value | Odds ratio (95%CI) | P value |
| Lean mass index | 0.93 (0.79, 1.10) | 0.4 | 0.91 (0.77, 1.08) | 0.3 |
| Fat mass index | 1.23 (1.04, 1.45) | 0.01 | 1.24 (1.04, 1.47) | 0.02 |

[a]Assessment of body composition measures has been described in detail by Bann et al[1]

[b] Model 1: includes sex, lean mass index and fat mass index

[c] Model 2: model 1 + education at age 26, occupational class at age 53 and the following covariates (assessed at age 60-64): height, cigarette smoking status, physical activity and symptoms of anxiety and depression

**Table S5: Odds Ratios (OR) of back pain at each age per 1 kg/m$^2$ increases in BMI and 1 cm increases in waist circumference at the same age estimated from multilevel logistic models (12 326 observations nested within 3426 individuals)**

| | BMI | | | |
|---|---|---|---|---|
| | Model 1[a] | | Model 2[b] | |
| Per kg/m$^2$ BMI at: | OR (95% CI) | P value | OR (95% CI) | P value |
| 36y | 1.03 (1.00,1.07) | 0.04 | 1.03 (1.00,1.06) | 0.05 |
| 43y | 1.02 (1.00,1.05) | 0.06 | 1.03 (1.00,1.05) | 0.05 |
| 53y | 1.04 (1.01,1.06) | 0.002 | 1.04 (1.01,1.06) | 0.002 |
| 60-64y | 1.06 (1.03,1.08) | <0.001 | 1.05 (1.03,1.08) | <0.001 |
| 68y | 1.02 (0.99,1.04) | 0.2 | 1.02 (0.99,1.04) | 0.2 |
| | Waist circumference | | | |
| | Model 1[a] | | Model 2[b] | |
| Per cm WC at: | OR (95% CI) | P value | OR (95% CI) | P value |
| 36y | 1.014 (1.004,1.023) | 0.005 | 1.011 (1.001,1.020) | 0.03 |
| 43y | 1.011 (1.002,1.020) | 0.01 | 1.008 (0.999,1.017) | 0.07 |
| 53y | 1.013 (1.005,1,021) | 0.002 | 1.011 (1.002,1,019) | 0.01 |
| 60-64y | 1.019 (1.009,1.030) | <0.001 | 1.016 (1.006,1.026) | 0.002 |
| 68y | 1.009 (0.999,1.019) | 0.07 | 1.006 (0.996,1.015) | 0.3 |

*Note.* BMI: body mass index; OR: Odds Ratio; y: years

[a] Model 1: Includes age as a categorical variable, BMI or WC and an age by BMI or age by WC interaction (as appropriate), adjusted for sex (as there was no evidence of a sex by BMI (p=0.5) or sex by WC (p=0.7) interaction)

[b] Model 2: Includes age as a categorical variable, BMI or WC and an age by BMI or age by WC interaction (as appropriate), adjusted for sex, education, occupational class and time-varying covariates (height, cigarette smoking status, physical activity and symptoms of anxiety and depression)



Muthuri S, *et al. BMJ Open* 2020; 10:e039197. doi: 10.1136/bmjopen-2020-039197

**Table S6: Odds Ratios (OR) of back pain at each age per 1 kg/m$^2$ increase in BMI and 1 cm increase in waist circumference at the previous age estimated from multilevel logistic models (8 595 observations nested within 3044 individuals)**

| | | BMI | | | |
|---|---|---|---|---|---|
| | | Model 1[a] | | Model 2[b] | |
| Per kg/m$^2$ BMI at age: | Back pain at age: | OR (95% CI) | P value | OR (95% CI) | P value |
| 36y | 43y | 1.02 (0.99, 1.05) | 0.1 | 1.02 (0.99, 1.05) | 0.2 |
| 43y | 53y | 1.04 (1.02, 1.07) | 0.002 | 1.04 (1.02, 1.07) | 0.004 |
| 53y | 60-64y | 1.06 (1.03, 1.09) | <0.001 | 1.05 (1.02, 1.08) | <0.001 |
| 60-64y | 68y | 1.02 (0.99, 1.05) | 0.2 | 1.02 (0.99, 1.05) | 0.3 |
| | | Waist circumference | | | |
| | | Model 1[a] | | Model 2[b] | |
| Per cm WC at age: | Back pain at age: | OR (95% CI) | P value | OR (95% CI) | P value |
| 36y | 43y | 1.012 (1.002,1.021) | 0.01 | 1.009 (0.999, 1.018) | 0.07 |
| 43y | 53y | 1.015 (1.006, 1.025) | 0.001 | 1.013 (1.004, 1.022) | 0.005 |
| 53y | 60-64y | 1.019 (1.009, 1.030) | <0.001 | 1.016 (1.005, 1.026) | 0.003 |
| 60-64y | 68y | 1.007 (0.996, 1.019) | 0.2 | 1.004 (0.993, 1.015) | 0.5 |

*Note.* BMI: body mass index; SD: Standard Deviation; OR: Odds Ratio; y: years

[a] Model 1: Includes age as a categorical variable, BMI or WC and an age by BMI or age by WC interaction (as appropriate), adjusted for sex (as there was no evidence of a sex by BMI (p=0.7) or sex by WC (p=0.9) interaction)

[b] Model 2: Includes age as a categorical variable, BMI or WC and an age by BMI or age by WC interaction (as appropriate), adjusted for sex, education, occupational class and time-varying covariates (height, cigarette smoking status, physical activity and symptoms of anxiety and depression)



Muthuri S, *et al. BMJ Open* 2020; 10:e039197. doi: 10.1136/bmjopen-2020-039197

## References

1. Bann D, Kuh D, Wills AK, et al. Physical activity across adulthood in relation to fat and lean body mass in early old age: Findings from the Medical Research Council National Survey of Health and Development, 1946–2010. *Am J Epidemiol.* 2014;179:1197-207.
