# Supplementary material for: Do the associations of body mass index and waist circumference with back pain change as people age? 32 years of follow-up in a British birth cohort
Source: BMJ Open. 2020 Dec 12;10(12):e039197. doi: 10.1136/bmjopen-2020-039197 (PMC7735102; doi:10.1136/bmjopen-2020-039197)
Supplement: Author's manuscript [file bmjopen-2020-039197.draft_revisions.pdf]

BMJ Open is committed to open peer review. As part of this commitment we make the peer review history of every article we publish publicly available.

When an article is published we post the peer reviewers' comments and the authors' responses online. We also post the versions of the paper that were used during peer review. These are the versions that the peer review comments apply to.

The versions of the paper that follow are the versions that were submitted during the peer review process. They are not the versions of record or the final published versions. They should not be cited or distributed as the published version of this manuscript.

BMJ Open is an open access journal and the full, final, typeset and author-corrected version of record of the manuscript is available on our site with no access controls, subscription charges or pay-per-view fees (http://bmjopen.bmj.com).

If you have any questions on BMJ Open's open peer review process please email info.bmjopen@bmj.com

BMJ Open

Do the associations of body mass index and waist circumference with back pain change as people age? 32 years of follow-up in a British birth cohort

Journal:	*BMJ Open*
Manuscript ID	bmjopen-2020-039197
Article Type:	Original research
Date Submitted by the Author:	07-Apr-2020

Complete List of Authors:	Muthuri, Stella; UCL, MRC Unit for Lifelong Health and Ageing<br>Cooper, Rachel; Manchester Metropolitan University, Sport and Exercise Sciences<br>Kuh, Diana; UCL, MRC Unit for Lifelong Health and Ageing<br>Hardy, Rebecca; UCL, CLOSER, UCL Institute of Education

Keywords:	Back pain < ORTHOPAEDIC & TRAUMA SURGERY, EPIDEMIOLOGY, PUBLIC HEALTH

SCHOLARONE™ Manuscripts

I, the Submitting Author has the right to grant and does grant on behalf of all authors of the Work (as defined in the below author licence), an exclusive licence and/or a non-exclusive licence for contributions from authors who are: i) UK Crown employees; ii) where BMJ has agreed a CC-BY licence shall apply, and/or iii) in accordance with the terms applicable for US Federal Government officers or employees acting as part of their official duties; on a worldwide, perpetual, irrevocable, royalty-free basis to BMJ Publishing Group Ltd ("BMJ") its licensees and where the relevant Journal is co-owned by BMJ to the co-owners of the Journal, to publish the Work in this journal and any other BMJ products and to exploit all rights, as set out in our licence.

The Submitting Author accepts and understands that any supply made under these terms is made by BMJ to the Submitting Author unless you are acting as an employee on behalf of your employer or a postgraduate student of an affiliated institution which is paying any applicable article publishing charge ("APC") for Open Access articles. Where the Submitting Author wishes to make the Work available on an Open Access basis (and intends to pay the relevant APC), the terms of reuse of such Open Access shall be governed by a Creative Commons licence – details of these licences and which Creative Commons licence will apply to this Work are set out in our licence referred to above.

Other than as permitted in any relevant BMJ Author's Self Archiving Policies, I confirm this Work has not been accepted for publication elsewhere, is not being considered for publication elsewhere and does not duplicate material already published. I confirm all authors consent to publication of this Work and authorise the granting of this licence.

For peer review only

Do the associations of body mass index and waist circumference with back pain change as people age? 32 years of follow-up in a British birth cohort

Stella G Muthuri[1], Rachel Cooper[2*], Diana Kuh[1], Rebecca Hardy[3]

[1] MRC Unit for Lifelong Health and Ageing at UCL, London, UK

[2] Musculoskeletal Science and Sports Medicine Research Centre, Department of Sport and Exercise Sciences, Manchester Metropolitan University, Manchester, UK

[3] CLOSER, UCL Institute of Education, University College London, London, UK

*** Corresponding author:** Rachel Cooper, Department of Sport and Exercise Sciences, All Saints Building, Manchester Metropolitan University, Manchester, M15 6BH, UK

Email: r.cooper@mmu.ac.uk

Word count (main text): 2850

Key words: back pain; obesity; adiposity; birth cohort; ageing

Contributors

SGM and RH had full access to all the data in the study and take full responsibility for the integrity and the accuracy of data analysis. SGM, DK, and RC conceived the idea for this study; SGM, DK, RH and RC contributed to the development of the study objectives; DK, RH and RC acquired the data; SGM and RH analysed the data; SGM and RH drafted the manuscript; all authors contributed to the manuscript's critical revision and provided final approval of the version to be published.

For peer review only - http://bmjopen.bmj.com/site/about/guidelines.xhtml

Abstract

Objectives: To investigate whether associations of body mass index (BMI) and waist circumference (WC) with back pain change with age and extend into later life.

Design: British birth cohort study

Setting: England, Scotland and Wales

Participants: Up to 3426 men and women from the MRC National Survey of Health and Development

Primary outcome measures: Back pain was self-reported at ages 36, 43, 53, 60-64 and 68 years. Mixed-effects logistic regression models were fitted to test the repeated cross-sectional and longitudinal associations of nurse-assessed BMI and WC with back pain and whether these associations varied by age.

Results: Higher BMI was consistently associated with increased odds of back pain at all ages, except at age 68, but was stronger at age 60-64 than at any other age. Sex-adjusted odds ratios of back pain per 1 standard deviation increase in BMI were: 1.13 (95% CI 1.01, 1.26), 1.11 (95% CI 1.00, 1.23), 1.17 (95% CI 1.05, 1.30), 1.31 (95% CI 1.15, 1.48) and 1.08 (95% CI 0.95, 1.24) at ages 36, 43, 53, 60-64 and 68-69, respectively. Similar patterns of associations were observed for WC. These associations were maintained when potential confounders, including education, occupational class, height, cigarette smoking status, physical activity and symptoms of anxiety and depression were accounted for. BMI showed stronger associations than WC in models including both measures.

Conclusions: These findings demonstrate that higher BMI is a persistent risk factor for back pain across adulthood. This highlights the potential lifelong consequences of the rising levels of obesity on back pain and outcomes these precipitate including disability and functional limitations in later life.

For peer review only - http://bmjopen.bmj.com/site/about/guidelines.xhtml

Article summary

Strengths and limitations of this study

- The availability of data on back pain, body mass index (BMI) and waist circumference (WC) prospectively ascertained over 32 years of follow-up in a large representative population-based sample is a key and unique strength of this study.
- This allowed us to formally test whether the cross-sectional and prospective relationships of BMI and WC with back pain change with age, extend into later life and are independent of each other so addressing important gaps in our current understanding.
- Back pain was not assessed in exactly the same way at different time points across adulthood, timing of onset of pain was not recorded and severity and chronicity were not distinguished but despite this we were able to show that associations were largely consistent across adulthood.
- Residual confounding cannot be fully ruled out as a potential explanation of our findings however, the risk of this has been minimised by taking account of a wide range of potential confounders measured prospectively across adulthood.
- Cross-sectional associations between body size and back pain may be explained by reverse causality, but we also investigated longitudinal associations between BMI and back pain and our conclusions remained unchanged.

For peer review only

For peer review only - http://bmjopen.bmj.com/site/about/guidelines.xhtml

Introduction

Back pain is one of the most commonly reported musculoskeletal disorders and was recently ranked as the number one cause of disability in most countries worldwide.[1] As it is a major cause of activity limitation and functional decline in later life[2-8] the fact that its prevalence is projected to increase as population ageing continues is a considerable cause for concern. As a result, there have been calls to intensify research efforts to address the public health challenge of the burden of low back pain.[9]

Obesity, usually indicated by high body mass index (BMI), has been identified as an important risk factor for back pain in systematic reviews.[10-12] However, the majority of existing studies have examined cross-sectional associations at a single time-point, often in early or mid-adulthood[10, 13-17] and/or are in occupational-based cohorts.[10] There are few studies on obesity and back pain in older populations. This is despite evidence to suggest that the aetiology of back pain may change with age as degenerative back disorders become increasingly common; these disorders may have different risk factors to conditions that precipitate back pain earlier in life. This is coupled with reduced pain-modulatory capacity occurring with advancing age partly explaining increases in persistent and disabling pain conditions among older adults.[18] In one study of older populations (age >50 years) from nine countries, high BMI was associated with increased odds of back pain in European countries (Poland, Russia, Finland, Spain) and South Africa.[19] However, whether or not the associations found among these older adults were weaker, due to potential changes in the underlying aetiology of back pain, or of similar strength to those that would have been observed in these populations if they had been assessed at younger ages could not be established. To our knowledge, no study has formally investigated whether the association

For peer review only - http://bmjopen.bmj.com/site/about/guidelines.xhtml

between BMI and back pain changes with age; thus whether or not obesity remains a suitable target for intervention for the prevention of back pain into later life remains to be established.

Higher central adiposity, as well as higher BMI, has been related to increased risk of back pain[12-17, 20] and may be more indicative of inflammatory processes due to increases in fat mass. Despite this, few studies[20] have compared the strength of associations between BMI and abdominal adiposity or tested whether there is any additional effect of abdominal adiposity over and above BMI as a general marker of total adiposity. This could provide new aetiological insights to help inform the development of interventions to prevent or alleviate back pain.

The MRC National Survey of Health and Development (NSHD), the oldest of the British birth cohort studies, has assessed BMI, waist circumference (WC) and back pain at multiple time points across adulthood from midlife up to age 69. It therefore provides a unique opportunity to address important research gaps by examining whether the cross-sectional and prospective relationships of BMI and WC with back pain change with age, extend into later life and are independent of each other.

Subjects and Methods

The NSHD is a socially stratified sample of 5362 single, legitimate births that occurred in England, Wales and Scotland in one week of March 1946. To date participants have been assessed on 24 occasions up to age 69.[21-23] All waves of data collection have complied with ethical standards. Ethical approval for the most recent data collection at age 68-69 was obtained from the Queen Square Research Ethics Committee (14/LO/1073) and the Scotland

For peer review only

A Research Ethics Committee (14/SS/1009). All methods were carried out in accordance with the relevant guidelines and regulations and written informed consent was obtained.

Patient and public involvement

Over the 73 years of this study, the research has increasingly involved participants, in line with changing norms about conducting cohort studies. Participant involvement includes receiving personal letters from the research team as required, and invitations to participate in birthday celebrations, public engagement activities and focus groups to discuss future data collections. When piloting new questionnaires and assessments, including those used in these analyses, patients from general practices and the University College London Hospitals Patient Public Involvement group were recruited and asked to provide feedback which was taken into account when designing the mainstage fieldwork.

Back pain assessment

Back pain was ascertained during nurse interviews at ages 36, 43, 53 and 60-64 and in a postal questionnaire at age 68. At all ages except age 68, participants were asked whether they had sciatica, lumbago, or recurring/severe backache all or most of the time (ever at ages 36 and 43 and in the previous 12 months at ages 53 and 60-64). At age 68, participants were asked whether they had experienced any ache or pain in the previous month which had lasted for one day or longer, not including pain occurring during the course of a feverish illness such as flu. Those who responded positively were asked to shade the location of their pain using a four-view body manikin. Those who shaded any back site were classified as having back pain for the purposes of these analyses.

Anthropometric measurements

For peer review only - http://bmjopen.bmj.com/site/about/guidelines.xhtml

Height, weight and waist circumference (taken at the midpoint between the costal margin and iliac crest) were measured by nurses using standardised protocols at ages 36, 43, 53, 60-64 and 69 years. BMI was calculated at each age as weight (kg)/height (m)^2. BMI and waist circumference at each age were sex-standardised (to a mean of 0 and standard deviation (SD) of 1); our units of analysis for BMI and waist circumference are both 1SD to facilitate comparisons of effect sizes across age and sex.

Fat and lean mass at 60-64 years

Measures of body composition were obtained in participants who attended one of six Clinical Research facilities when aged 60-64 years using a QDR 4500 Discovery DXA scanner (Hologic Inc, Bedford, MA, USA) whilst the individuals were in a supine position. Whole body fat mass was measured and whole body lean mass was calculated as total mass minus fat and bone mass. Mass from the head was excluded and measures were converted to kg. Full details are provided elsewhere.[24] Fat mass index (kg/m^2) and lean mass index (kg/m^2) were calculated by dividing the measures of mass by height-squared.

Covariates

Covariates representing different domains of the biopsychosocial model of pain were selected *a priori*.[25] This included variables that have previously been identified as key risk factors for back pain.[20, 26, 27] These were sex, educational attainment at age 26, occupational class at age 53, measures of height, smoking status, physical activity, and symptoms of anxiety and depression, which were available at ages 36, 43, 53, 60-64 and 68-69 and so included as time varying covariates.

For peer review only - http://bmjopen.bmj.com/site/about/guidelines.xhtml

For peer review only

The highest education level achieved by age 26 was grouped into no qualifications, up to O-level or equivalent, or A-level or equivalent and above. Own occupation at age 53 was categorised according to the Registrar General's social classification into three groups: high (I or II: professional, managerial or technical); middle (IIINM skilled non-manual or IIIM: skilled manual); low (IV or V: partly skilled or unskilled manual).

Smoking status was assessed by self-report at each wave and categorised as never-, ex and current smoker. Participation in sports, vigorous leisure activities or exercise was assessed at each wave and participants were grouped as inactive, moderately active (1-4 times/month) or regularly active (≥5 times/month).[28]

Symptoms of anxiety and depression were also assessed at all five waves. At age 36, a shortened version of the Present State Examination[29] was used at a nurse interview and the total score was recoded to a binary variable using the recommended threshold for caseness of 5 or more. At age 43, participants completed the 18-item Psychiatric Symptom Frequency (PSF) scale[30] and the total score was calculated and then dichotomised into absence of symptoms (PSF score <23) and presence of symptoms (PSF score ≥23). At ages 53, 60-64 and 68/69 the 28-item General Health Questionnaire (GHQ-28)[31] was self-administered and scores of all items were summed and further dichotomised using a recommended threshold for caseness of 5 or more.

Statistical analysis

Multilevel mixed-effects logistic regression models with a random intercept were used to test associations between BMI and back pain with measurement occasion nested within individuals. These models allow for the correlation between measurements on the same

For peer review only - http://bmjopen.bmj.com/site/about/guidelines.xhtml

individual. We fitted measurement wave as a categorical variable with age 36 as the reference category and adjusted for sex. Interactions between wave and sex were tested in the model however, these were not statistically significant and so were removed in subsequent models. We then tested associations of BMI with back pain across adulthood by fitting body size measures as time varying covariates. Formal tests of sex interaction were performed by including sex by BMI interaction terms in models. Deviations from linearity were assessed by including quadratic terms for body size but no evidence of this was found. To assess whether the association of BMI with back pain changed with age, separate models were fitted that included interactions between wave and BMI. Models were then additionally adjusted for potential confounding variables. Analyses were repeated replacing BMI with WC and then a final confounder adjusted model was run in which BMI and WC were included together. All models included the maximum number of participants, which was those participants with a valid measure of back pain, BMI, WC and all covariates for at least one age (n=3426) (see table S1 for number of participants who contributed data at each wave).

We then repeated the same modelling approach to assess prospective associations between body size and back pain. To achieve this, we used back pain at 43, 53, 60-64 and 68 as outcomes and related them to body size at the prior age (i.e. at ages 36, 43, 53 and 60-64). These models included a sample size of 3044.

Finally, in the sub-sample with body composition measures at age 60-64 (n=1186) supplementary analyses using multiple regression models, assessed the associations between fat mass index and lean mass index at 60-64 years and back pain at 68 years. Model 1 included sex and both body composition measures and model 2 added the same covariates used in previous models.

For peer review only - http://bmjopen.bmj.com/site/about/guidelines.xhtml

All analyses were performed using STATA version 14.1.

Results

The characteristics of the study sample are shown in Table 1 (and Table S2). Overall, the percentage reporting back pain varied from 17.9% and 32.3% and was higher in women than men at every age (Table 1).

In sex-adjusted models, higher BMI was associated with increased odds of back pain at every age (Table 2). Sex-adjusted odds ratios (ORs) of back pain per 1SD increase in BMI were 1.13 (95% CI 1.01, 1.26), 1.11 (95% CI 1.00, 1.23), 1.17 (95% CI 1.05, 1.30), 1.31 (95% CI 1.15, 1.48) and 1.08 (95% CI 0.95, 1.24) at ages 36, 43, 53, 60-64 and 68-69, respectively. Adjustment for potential confounders had minimal impact on these ORs (Table 2). There was some evidence to suggest that the association was strongest at age 60-64 (age 60-64 wave by BMI interaction: p=0.07) but there was no evidence of a difference in association between age 36 and any other age (wave by BMI interactions p>0.5) (Supplementary Table S3). There was also evidence to suggest that the association at age 60-64 was stronger than the associations at 53 (p=0.03) and 68-69 (p=0.03).

A similar, albeit less variable, pattern of association was observed for WC, with no evidence that the association at age 60-64 was stronger than at other ages (wave by BMI interaction: p=0.2) (Table 2, Supplementary Table S3). Adjustment for potential confounders attenuated all ORs somewhat, and to a greater extent than the ORs for BMI (Table 2).

For peer review only - http://bmjopen.bmj.com/site/about/guidelines.xhtml

Including both BMI and WC in the same fully adjusted model with all interaction terms led to inflated standard errors. We therefore included only the age 60-64 wave by BMI and the age 60-64 wave by WC interaction, finding only evidence of the interaction with BMI. Our final fully adjusted model thus included only the interaction with BMI; the association of BMI with back pain (OR per SD (95% CI): 1.11 (0.99, 1.24) at 36, 43, 53 and 68-69y and 1.28 (1.09,1.50) at 60-64y) was stronger than that for WC (OR per SD (95% CI): 1.02 (0.91,1.13)) at all ages.

Findings for both BMI and WC were very similar when relating the body size measure at the prior age to subsequent back pain (Table 3). In supplementary analyses (n=1186), higher fat mass index was associated with higher odds of back pain at age 68 (OR per SD (95% CI): 1.23 (1.04, 1.45)) in a model adjusting for sex and lean mass index, but no association was observed for lean mass index (Supplementary Table S4). The association with fat mass index was maintained after adjustment for other potential confounders.

Discussion

In a large nationally representative British birth cohort higher BMI and WC were consistently associated with increased odds of back pain between ages 36 and 68. These associations were maintained after adjustment for confounders and, BMI remained more strongly related to back pain, particularly at age 60-64 years, than WC in a model including both variables. Findings were similar when prospective associations were investigated for back pain between 43 and 68.

Our findings are consistent with previous studies which have reported associations between overweight and obesity, indicated by high BMI, and increased odds of back pain.[10]

For peer review only - http://bmjopen.bmj.com/site/about/guidelines.xhtml

Findings from cross-sectional studies also suggest positive associations between higher WC and low back pain in women, but not in men.[12-17] These previous studies have sampled participants across a range of different ages (15 years and older) and although they have adjusted for age, none has examined whether associations of BMI and WC with back pain vary with age or whether associations remain into older age. Our study shows persisting associations of BMI and WC with back pain until age 68 years with little evidence of variation with age. The slightly stronger association at age 60-64 needs to be interpreted with caution as this may be due to the differences in how the outcome was assessed at different ages. However, the question used at 60-64 was also used at age 53 and this shows an association consistent with other ages. In addition, we are confident that the association does persist into older age because associations at each age were fairly constant despite the differences in method of outcome ascertainment.

Another key finding is that BMI exhibited a stronger association than WC in a model including both measures. This suggests that mechanisms specifically related to central adiposity do not fully underlie the associations observed. This contrasts with a previous study in young adults (age 24-39 years) which found that WC but not BMI remained associated with back pain in mutually adjusted models in women only.[13] Our findings in relation to fat and lean mass suggest that associations with BMI may be driven by whole body fat mass, rather than abdominal fat mass specifically. In testing these associations we are addressing the call made in a recent systematic review for further high-quality studies on the relationships of body composition with pain.[32] Our findings are consistent with a prospective study[12] included in this review that found evidence that fat mass was associated with low back pain intensity and disability in both sexes. Likewise, a cross-sectional study found that higher fat mass (independent of lean mass) was associated with

For peer review only - http://bmjopen.bmj.com/site/about/guidelines.xhtml

higher levels of low back pain intensity and disability but found no relationship with lean mass after adjusting for fat mass.[11] The stronger associations with BMI than WC may point to the importance of mechanical effects, with increased BMI resulting in higher compressive force on the spine during activity.[10] However, we cannot rule out the fact that chronic inflammatory or metabolic pathways may also be relevant. For example, atherosclerosis and chronic inflammation, which are linked to obesity, have also been associated with disc degeneration and low back pain.[33, 34]

A key strength of our study is the large representative population-based sample of adults with assessments of back pain and measured height, weight and waist circumference at five time points over 32 years of follow-up. Although back pain was not assessed in exactly the same way at different time points across adulthood and, severity and chronicity were not distinguished we were able to show that associations are consistent across adulthood and persist into old age. Another strength is the availability of data on a wide range of potential confounders measured prospectively across adulthood that allowed us to adjust for a range of time-varying covariates. While residual confounding cannot be fully ruled out, the risk of this has been minimised. It is acknowledged that any cross-sectional associations between body size and back pain may be explained by reverse causality, but we also investigated associations between back pain and BMI measured at a prior age and findings remained unchanged. Finally, our study population comprised Caucasian men and women born in Britain in 1946. While this allows us to rule out confounding by age or ethnicity as potential explanations of our findings, it may limit their generalisability. Further research is thus necessary to consider the implications of these findings for more ethnically diverse cohorts and also more recently born cohorts, the latter who are likely to have spent more of their lives overweight or obese.[35]

For peer review only - http://bmjopen.bmj.com/site/about/guidelines.xhtml

For peer review only

In summary, our findings suggest that there is a consistent relationship between higher adiposity and back pain across adulthood. Our results also show that WC does not add further information once BMI has been accounted for in this relationship. This underscores the importance of primary and secondary interventions to prevent excessive weight gain and reduce overweight and obesity across the whole of adulthood in order to help prevent back pain and its disabling consequences as individuals age.

For peer review only

For peer review only - http://bmjopen.bmj.com/site/about/guidelines.xhtml

Acknowledgements

The authors are grateful to NSHD study members for their continuing participation in the study. We also thank members of the NSHD scientific and data collection teams.

Funding statement

This work was supported by the UK Medical Research Council (Programme codes: MC_UU_12019/4 and MC_UU_12019/2). RH is Director of the CLOSER consortium which is supported by funding from the Economic and Social Research Council (ESRC) and the Medical Research Council (MRC). An initial grant for CLOSER was awarded in 2012 and extended, by the ESRC, in 2017 (award reference: ES/K000357/1).

Competing interests

The authors declare no financial or non-financial competing interests

Data sharing statement

Data used in this publication are available to bona fide researchers upon request to the NSHD Data Sharing Committee via a standard application procedure. Further details can be found at http://www.nshd.mrc.ac.uk/data. doi: 10.5522/NSHD/Q101; doi: 10.5522/NSHD/Q102; 10.5522/NSHD/Q103

For peer review only

For peer review only - http://bmjopen.bmj.com/site/about/guidelines.xhtml

References

1. GBD 2015 Disease and Injury Incidence and Prevalence Collaborators. Global, regional, and national incidence, prevalence, and years lived with disability for 310 diseases and injuries, 1990-2015: a systematic analysis for the Global Burden of Disease Study 2015. *Lancet* 2016;388:1545-602.

2. Dionne CE, Dunn KM, Croft PR. Does back pain prevalence really decrease with increasing age? A systematic review. *Age Ageing* 2006;35:229-34.

3. Docking RE, Fleming J, Brayne C, et al. Epidemiology of back pain in older adults: prevalence and risk factors for back pain onset. *Rheumatology*. 2011;50:1645-53.

4. Leveille SG, Bean J, Ngo L, et al. The pathway from musculoskeletal pain to mobility difficulty in older disabled women. *Pain.* 2007;128:69-77.

5. Patel KV, Guralnik JM, Dansie EJ, et al. Prevalence and impact of pain among older adults in the United States: findings from the 2011 National Health and Aging Trends Study. *Pain.* 2013:154:2649-57.

6. Marshall LM, Litwack-Harrison S, Cawthon PM, et al. A prospective study of back pain and risk of falls among older community-dwelling women. *J Gerontol Med Sci.* 2016:71:1177-83.

7. O'Sullivan K, Kennedy N, Purtill H, et al. Understanding pain among older persons: Part 1-the development of novel pain profiles and their association with disability and quality of life. *Age Ageing.* 2017;46:46-51.

8. Kennedy N, O'Sullivan K, Hannigan A, et al. Understanding pain among older persons: Part 2—the association between pain profiles and healthcare utilisation. *Age Ageing.* 2017;46:51-6.

9. Hartvigsen J, Hancock MJ, Kongsted A. et al. What low back pain is and why we need to pay attention. *Lancet* 2018;391:2356-67.

For peer review only - http://bmjopen.bmj.com/site/about/guidelines.xhtml

10. Shiri R, Karppinen J, Leino-Arjas P, et al. The association between obesity and low back pain: a meta-analysis. *Am J Epidemiol.* 2010;171:135-54.

11. Urquhart DM, Berry P, Wluka AE, et al. 2011 Young Investigator Award winner: Increased fat mass is associated with high levels of low back pain intensity and disability. *Spine (Phila Pa 1976).* 2011;36:1320-5.

12. Hussain SM, Urquhart DM, Wang Y, et al. Fat mass and fat distribution are associated with low back pain intensity and disability: results from a cohort study. *Arthritis Res Ther.* 2017;19:26.

13. Shiri R, Solovieva S, Husgafvel-Pursiainen K, et al. The association between obesity and the prevalence of low back pain in young adults: the Cardiovascular Risk in Young Finns Study. *Am J Epidemiol.* 2008;167:1110-9

14. Han TS, Schouten JS, Lean ME, et al. The prevalence of low back pain and associations with body fatness, fat distribution and height. *Int J Obes Relat Metab Disord.* 1997;21:600-97.

15. Toda Y, Segal N, Toda T, et al. Lean body mass and body fat distribution in participants with chronic low back pain. *Arch Intern Med.* 2000;160:3265-9.

16. Lean ME, Han TS, Seidell JC. Impairment of health and quality of life in people with large waist circumference. *Lancet.* 1998;351:853-6.

17. Frilander H, Solovieva S, Mutanen P, et al. Role of overweight and obesity in low back disorders among men: a longitudinal study with a life course approach. *BMJ Open.* 2015;5:e007805.

18. Gagliese L. Pain and aging: The emergence of a new subfield of pain research. *J Pain.* 2009;10:343-53.

19. Koyanagi A, Stickley A, Garin N, et al. The association between obesity and back pain in nine countries: a cross-sectional study. *BMC Public Health.* 2015;15:123.

For peer review only - http://bmjopen.bmj.com/site/about/guidelines.xhtml

20. Heuch I, Heuch I, Hagen K, et al. Association between body height and chronic low back pain: a follow-up in the Nord-Trøndelag Health Study. *BMJ Open.* 2015;5:e006983.

21. Kuh D, Pierce M, Adams J, et al. Cohort profile: updating the cohort profile for the MRC National Survey of Health and Development: a new clinic-based data collection for ageing research. *Int J Epidemiol.* 2011;40:e1-9.

22. Kuh D, Wong A, Shah I, et al. The MRC National Survey of Health and Development reaches age 70: maintaining participation at older ages in a birth cohort study. *Eur J Epidemiol.* 2016;31:1135-47.

23. Wadsworth M, Kuh D, Richards M, et al. Cohort Profile: The 1946 National Birth Cohort (MRC National Survey of Health and Development). *Int J Epidemiol.* 2006;35:49-54.

24. Bann D, Kuh D, Wills AK, et al. Physical activity across adulthood in relation to fat and lean body mass in early old age: findings from the Medical Research Council National Survey of Health and Development, 1946-2010. *Am J Epidemiol.* 2014;179:1197-207.

25. Gatchel RJ, Peng YB, Peters ML, et al. The biopsychosocial approach to chronic pain: Scientific advances and future directions. *Psychol Bull.* 2007;133:581-624.

26. Hoy D, Brooks P, Blyth F, et al. The epidemiology of low back pain. *Best Pract Res Clin Rheumatol.* 2010;24:769-81.

27. Kherad M, Rosengren BE, Hasserius R, et al. Risk factors for low back pain and sciatica in elderly men-the MrOS Sweden study. *Age Ageing*. 2017;46:64-71.

28. Elhakeem A, Cooper R, Bann D, et al. Birth weight, school sports ability, and adulthood leisure-time physical activity. *Med Sci Sports Exerc.* 2017;49:64-70.

29. Wing J, Cooper J, Sartorius N. The measurement and classification of psychiatric symptoms. Cambridge: Cambridge University Press; 1974.

For peer review only - http://bmjopen.bmj.com/site/about/guidelines.xhtml

30. Lindelow M, Hardy R, Rodgers B. Development of a scale to measure symptoms of anxiety and depression in the general UK population: the psychiatric symptom frequency scale. *J Epidemiol Community Health.* 1997;51:549-57.

31. Goldberg DP, Hillier VF. A scaled version of the General Health Questionnaire. *Psychol Med*. 1979;9:139-45.

32. Walsh TP, Arnold JB, Evans AM, et al. The association between body fat and musculoskeletal pain: A systematic review and meta-analysis. *BMC Musculoskelet Disord.* 2018;19:233.

33. Kauppila LI. Atherosclerosis and disc degeneration/low-back pain-a systematic review. *Eur J Vasc Endovasc Surg.* 2009;37:661-70.

34. Ruiz-Fernández C, Francisco V, Pino J, et al. Molecular relationships among obesity, inflammation and intervertebral disc degeneration: Are adipokines the common link? *Int J Mol Sci.* 2019;20:E2030.

35. Johnson W, Li L, Kuh D, et al. How has the age-related process of overweight or obesity development changed over time? Co-ordinated analyses of individual participant data from five United Kingdom birth cohort. *PLoS Med.* 2015;12:e1001828.

For peer review only

For peer review only - http://bmjopen.bmj.com/site/about/guidelines.xhtml

Table 1: Characteristics of the MRC National Survey of Health and Development sample included in analysis [a]

		Male	Female	p-value[b]
**Back pain, N (%), at age:**				
36y				.08
	No	1283 (83.3)	1256 (80.9)	
	Yes	257 (16.7)	297 (19.1)	
43y				.06
	No	1106 (74.6)	1042 (71.5)	
	Yes	377 (25.4)	415 (28.6)	
53y				.06
	No	925 (69.4)	903 (66.0)	
	Yes	407 (30.6)	465 (34.0)	
60-64y				.06
	No	629 (72.1)	643 (68.1)	
	Yes	243 (27.9)	301 (31.9)	
68y				.02
	No	637 (74.1)	635 (69.2)	
	Yes	223 (25.9)	282 (30.8)	
**BMI (kg/m^2), mean (SD), at age:**				
36y		24.8 (3.3)	23.5 (4.1)	<.001
43y		25.7 (3.5)	25.1 (4.7)	<.001
53y		27.4 (4.1)	27.4 (5.4)	.85
60-64y		27.9 (4.1)	27.9 (5.5)	.99
69y		28.1 (4.5)	28.0 (5.6)	.93
**Waist circumference (cm), mean (SD), at age:**				
36y		89.5 (9.3)	76.9 (11.5)	<.001
43y		91.8 (9.9)	77.7 (11.2)	<.001
53y		97.7 (10.9)	85.8 (12.8)	<.001
60-64y		100.8 (11.1)	92.2 (13.0)	<.001
69y		100.9 (12.3)	92.1 (14.0)	<.001

Note. BMI: body mass index; WC: waist circumference; SD: Standard Deviation; y: years

[a] Maximum N=3426 (1723 males and 1703 females) (this includes participants with a valid measure of back pain, BMI, waist circumference and each covariate for at least one age). Number of participants contributing data at each age: 36y, N=3093; 43y, N=2940; 53y, N=2700; 60-64y, N=1816; 68-69y, N=1777 (see Supplementary table S1 for more details)

[b] p-values from formal tests of sex differences using chi-squared and t-tests as appropriate

For peer review only - http://bmjopen.bmj.com/site/about/guidelines.xhtml

Table 2: Odds Ratios (OR) of back pain at each age per 1 standard deviation increases in BMI and waist circumference at the same age estimated from multilevel logistic models (12 326 observations nested within 3426 individuals)

	BMI			
	Model 1[a]		Model 2[b]	
Per SD BMI at:	OR (95% CI)	P value	OR (95% CI)	P value
36y	1.13 (1.01 , 1.26)	0.03	1.12 (1.00,1.25)	0.05
43y	1.11 (1.00 , 1.23)	0.06	1.11 (1.00,1.23)	0.05
53y	1.17 (1.05 , 1.30)	0.003	1.17 (1.05,1.30)	0.003
63y	1.31 (1.15 , 1.48)	<0.001	1.30 (1.14,1.47)	<0.001
68y	1.08 (0.95 , 1.24)	0.2	1.07 (0.94,1.22)	0.3
	Waist circumference			
	Model 1[a]		Model 2[b]	
Per SD WC at:	OR (95% CI)	P value	OR (95% CI)	P value
36y	1.17 (1.05 , 1.31)	0.005	1.14 (1.02,1.27)	0.02
43y	1.12 (1.01 , 1.24)	0.03	1.08 (0.98,1.20)	0.1
53y	1.16 (1.05 , 1.29)	0.005	1.13 (1.02,1.25)	0.03
63y	1.27 (1.12 , 1.44)	<0.001	1.21 (1.07,1.38)	0.003
68y	1.12 (0.98 , 1.27)	0.1	1.07 (0.94,1.22)	0.3

Note. BMI: body mass index; SD: Standard Deviation; OR: Odds Ratio; y: years

[a] Model 1: adjusted for sex

[b] Model 2: adjusted for sex, education, occupational class and time-varying covariates (height, cigarette smoking status, physical activity and symptoms of anxiety and depression)

For peer review only - http://bmjopen.bmj.com/site/about/guidelines.xhtml

Table 3: Odds Ratios (OR) of back pain at each age per 1 standard deviation increase in BMI and waist circumference at the previous age estimated from multilevel logistic models (8 595 observations nested within 3044 individuals)

		BMI			
		Model 1[a]		Model 2[b]	
Per SD BMI/WC at age:	Back pain at age:	OR (95% CI)	P value	OR (95% CI)	P value
36y	43y	1.08 (0.97 , 1.20)	0.2	1.06 (0.96,1.18)	0.3
43y	53y	1.17 (1.05 , 1.31)	0.004	1.16 (1.04,1.29)	0.009
53y	63y	1.32 (1.15 , 1.51)	<0.001	1.29 (1.13,1.48)	<0.001
63y	68y	1.11 (0.96 , 1.28)	0.2	1.08 (0.94,1.25)	0.3
		Waist circumference			
		Model 1[a]		Model 2[b]	
		OR (95% CI)	P value	OR (95% CI)	P value
36y	43y	1.12 (1.01 , 1.24)	0.04	1.08 (0.97,1.21)	0.1
43y	53y	1.17 (1.05 , 1.30)	0.004	1.14 (1.02,1.26)	0.02
53y	63y	1.28 (1.12 , 1.47)	<0.001	1.23 (1.07,1.40)	0.003
63y	68y	1.10 (0.96 , 1.26)	0.2	1.06 (0.92,1.21)	0.4

Note. BMI: body mass index; SD: Standard Deviation; OR: Odds Ratio; y: years

[a] Model 1: adjusted for sex

[b] Model 2: adjusted for sex, education, occupational class and time-varying covariates (height, cigarette smoking status, physical activity and symptoms of anxiety and depression)

For peer review only

Supplementary information: Do the associations of body mass index and waist circumference with back pain change as people age? 32 years of follow-up in a British birth cohort

Stella G Muthuri, Rachel Cooper, Diana Kuh & Rebecca Hardy

Table S1: Number of MRC National Survey of Health and Development participants contributing data at each age and number with missing data[a]

Age	Total N contributing data at specified age (no. of males; no. of females)	N with data on back pain but missing data on BMI/WC at specified age	N with data on back pain and BMI/WC at specified age but missing data on covariates
36y	3093 (1540; 1553)	48	175
43y	2940 (1483; 1457)	52	259
53y	2700 (1332; 1368)	44	243
60-64y	1816 (872; 944)	29	390
68-69y	1777 (860; 917)	485	158

[a] A total of 3426 MRC NSHD participants were included in the main analyses. Participant's data could be included at a specified age if there was complete information on back pain, BMI, waist circumference and all covariates at that age
BMI: Body mass index; WC: waist circumference

For peer review only

Table S2: Characteristics of the MRC National Survey of Health and Development sample included in analysis [a]

	Male	Female
**Highest education level achieved by age 26y, N (%)**		
A-level/equivalent or above	706 (41.0)	463 (27.2)
O-level/equivalent or below	357 (20.7)	587 (34.5)
None	660 (38.3)	653 (38.3)
**Own occupational class at age 53y, N (%)**		
High (I/II)	853 (49.5)	593 (34.8)
Middle (IIINM/IIIM)	662 (38.4)	740 (43.5)
Low (IV/V)	208 (12.1)	370 (21.7)
**Time varying covariates[b]**		
**Height (cm), mean (SD), at age:**		
36y	175.4 (6.5)	162.4 (6.0)
43y	175.2 (6.6)	162.4 (6.2)
53y	174.7 (6.5)	161.6 (5.9)
60-64y	174.8 (6.5)	161.9 (5.9)
69y	174.0 (6.4)	160.7 (5.9)
**Smoking status, N (%), at age:**		
36y		
Current	530 (34.4)	524 (33.7)
Ex	617 (40.1)	518 (33.4)
Never	393 (25.5)	511 (32.9)
43y		
Current	460 (31.0)	417 (28.6)
Ex	522 (35.2)	373 (25.6)
Never	501 (33.8)	667 (45.8)
53y		
Current	323 (24.3)	315 (23.0)
Ex	544 (40.8)	391 (28.6)
Never	465 (34.9)	662 (48.4)
60-64y		
Current	97 (11.1)	105 (11.1)
Ex	410 (47.0)	322 (34.1)
Never	365 (41.9)	517 (54.8)
68-69y		
Current	76 (8.8)	65 (7.1)
Ex	411 (47.8)	314 (34.2)
Never	373 (43.4)	538 (58.7)
**Leisure time physical activity, N (%), at age:**		
36y		
Inactive	475 (30.8)	651 (41.9)
Less active	407 (26.4)	376 (24.2)

For peer review only - http://bmjopen.bmj.com/site/about/guidelines.xhtml

	Most active	658 (42.7)	526 (33.9)
43y			
	Inactive	714 (48.2)	805 (55.3)
	Less active	354 (23.9)	338 (23.2)
	Most active	415 (28.0)	314 (21.6)
53y			
	Inactive	627 (47.1)	689 (50.4)
	Less active	252 (18.9)	227 (16.6)
	Most active	453 (34.0)	452 (33.0)
60-64y			
	Inactive	557 (63.9)	585 (62.0)
	Less active	118 (13.5)	142 (15.0)
	Most active	197 (22.6)	217 (23.0)
68-69y			
	Inactive	491 (57.1)	520 (56.7)
	Less active	101 (11.7)	141 (15.4)
	Most active	268 (31.2)	256 (27.9)
**Symptoms of anxiety and depression, N(%), at age:**			
36y			
	No	1482 (96.2)	1420 (91.4)
	Yes	58 (3.8)	133 (8.6)
43y			
	No	1350 (91.0)	1233 (84.6)
	Yes	133 (9.0)	224 (15.4)
53y			
	No	1147 (86.1)	1035 (75.7)
	Yes	185 (13.9)	333 (24.3)
60-64y			
	No	765 (87.7)	739 (78.3)
	Yes	107 (12.3)	205 (21.7)
69y			
	No	781 (90.8)	764 (83.3)
	Yes	79 (9.2)	153 (16.7)

SD: Standard Deviation; y: years

[a] Maximum N=3426 (this includes participants with a valid measure of back pain, BMI, waist circumference and each covariate for at least one age)

[b] See number of participants with valid data at specified age in Supplementary Table S1

For peer review only

Table S3: Coefficients from the multilevel models for BMI and waist circumference with back pain across adulthood (N=3426)

	BMI			
	Model 1		Model 2[a]	
	Log-odds (SE)	P value	Log-odds (SE)	P value
BMI (per SD)	0.119 (0.071)	0.03	0.112 (0.056)	0.05
Sex (female)	0.221 (0.072)	0.002	0.500 (0.102)	<0.001
Age 43	0.683 (0.073)	<0.001	0.674 (0.074)	<0.001
Age 53	1.008 (0.074)	<0.001	0.993 (0.076)	<0.001
Age 60-64	0.869 (0.083)	<0.001	0.895 (0.087)	<0.001
Age 68	0.786 (0.084)	<0.001	0.876 (0.088)	<0.001
Age 43*BMI	-0.019 (0.071)	0.8	-0.009 (0.071)	0.9
Age 53*BMI	0.037 (0.072)	0.5	0.045 (0.072)	0.5
Age 60-64*BMI	0.148 (0.082)	0.07	0.148 (0.081)	0.07
Age 68*BMI	-0.040 (0.084)	0.6	-0.046 (0.084)	0.6

	Waist circumference			
	Model 1		Model 2[a]	
	Log-odds (SE)	P value	Log-odds (SE)	P value
WC (per SD)	0.160 (0.057)	0.005	0.129 (0.057)	0.02
Sex (female)	0.221 (0.071)	0.002	0.442 (0.102)	<0.001
Age 43	0.684 (0.073)	<0.001	0.674 (0.074)	<0.001
Age 53	1.010 (0.074)	<0.001	0.990 (0.076)	<0.001
Age 60-64	0.875 (0.083)	<0.001	0.896 (0.087)	<0.001
Age 68	0.787 (0.084)	<0.001	0.867 (0.088)	<0.001
Age 43*WC	-0.048 (0.072)	0.5	-0.049 (0.072)	0.5
Age 53*WC	-0.011 (0.073)	0.9	-0.009 (0.073)	0.9
Age 60-64*WC	0.076 (0.083)	0.4	0.063 (0.083)	0.5
Age 68*WC	-0.050 (0.084)	0.6	-0.058 (0.084)	0.5

Note. BMI: body mass index; WC: waist circumference; SE: standard error; SD: Standard Deviation;

[a] Model 2: model 1 + education, occupational class and time-varying covariates (height, cigarette smoking status, physical activity and symptoms of anxiety and depression)

For peer review only

Table S4: Associations between body composition measures[a] at age 60-64 and back pain at age 68 (n=1186)

	Model 1[b]		Model 2[c]	
Sex-standardised body composition measures	Odds ratio (95%CI)	P value	Odds ratio (95%CI)	P value
Lean mass index	0.93 (0.79, 1.10)	0.4	0.91 (0.77, 1.08)	0.3
Fat mass index	1.23 (1.04, 1.45)	0.01	1.24 (1.04, 1.47)	0.02

[a]Assessment of body composition measures has been described in detail by Bann et al[1]

[b] Model 1: includes sex, lean mass index and fat mass index

[c] Model 2: model 1 + education at age 26, occupational class at age 53 and the following covariates (assessed at age 60-64): height, cigarette smoking status, physical activity and symptoms of anxiety and depression

References

1. Bann D, Kuh D, Wills AK, et al. Physical activity across adulthood in relation to fat and lean body mass in early old age: Findings from the Medical Research Council National Survey of Health and Development, 1946–2010. *Am J Epidemiol.* 2014;179:1197-207.

For peer review only - http://bmjopen.bmj.com/site/about/guidelines.xhtml

STROBE 2007 (v4) Statement—Checklist of items that should be included in reports of *cohort studies*
Checklist completed for: Do the associations of body mass index and waist circumference with back pain change as people age? 32 years of follow-up in a British birth cohort

Section/Topic	Item #	Recommendation	Reported on page #
**Title and abstract**	1	(*a*) Indicate the study's design with a commonly used term in the title or the abstract	1; 2
		(*b*) Provide in the abstract an informative and balanced summary of what was done and what was found	2
**Introduction**			
Background/rationale	2	Explain the scientific background and rationale for the investigation being reported	4-5
Objectives	3	State specific objectives, including any prespecified hypotheses	5
**Methods**			
Study design	4	Present key elements of study design early in the paper	5-9
Setting	5	Describe the setting, locations, and relevant dates, including periods of recruitment, exposure, follow-up, and data collection	5-6
Participants	6	(*a*) Give the eligibility criteria, and the sources and methods of selection of participants. Describe methods of follow-up	5-9
		(*b*) For matched studies, give matching criteria and number of exposed and unexposed	n/a
Variables	7	Clearly define all outcomes, exposures, predictors, potential confounders, and effect modifiers. Give diagnostic criteria, if applicable	6-8
Data sources/ measurement	8*	For each variable of interest, give sources of data and details of methods of assessment (measurement). Describe comparability of assessment methods if there is more than one group	6-8
Bias	9	Describe any efforts to address potential sources of bias	8-9
Study size	10	Explain how the study size was arrived at	9, Table S1
Quantitative variables	11	Explain how quantitative variables were handled in the analyses. If applicable, describe which groupings were chosen and why	6-8, Table 1, Table S2
Statistical methods	12	(*a*) Describe all statistical methods, including those used to control for confounding	8-9
		(*b*) Describe any methods used to examine subgroups and interactions	8-9
		(*c*) Explain how missing data were addressed	8-9, table S1
		(*d*) If applicable, explain how loss to follow-up was addressed	n/a

For peer review only

		(*e*) Describe any sensitivity analyses	9
**Results**			
Participants	13*	(a) Report numbers of individuals at each stage of study—eg numbers potentially eligible, examined for eligibility, confirmed eligible, included in the study, completing follow-up, and analysed	5, 9
		(b) Give reasons for non-participation at each stage	5
		(c) Consider use of a flow diagram	See Stafford et al 2013
Descriptive data	14*	(a) Give characteristics of study participants (eg demographic, clinical, social) and information on exposures and potential confounders	Table 1 and Table S2
		(b) Indicate number of participants with missing data for each variable of interest	Table S1 and Table S2
		(c) Summarise follow-up time (eg, average and total amount)	n/a
Outcome data	15*	Report numbers of outcome events or summary measures over time	Tables
Main results	16	(*a*) Give unadjusted estimates and, if applicable, confounder-adjusted estimates and their precision (eg, 95% confidence interval). Make clear which confounders were adjusted for and why they were included	9-11, Tables 2-3
		(*b*) Report category boundaries when continuous variables were categorized	Tables 1-3
		(*c*) If relevant, consider translating estimates of relative risk into absolute risk for a meaningful time period	-
Other analyses	17	Report other analyses done—eg analyses of subgroups and interactions, and sensitivity analyses	9
**Discussion**			
Key results	18	Summarise key results with reference to study objectives	11
**Limitations**			
Interpretation	20	Give a cautious overall interpretation of results considering objectives, limitations, multiplicity of analyses, results from similar studies, and other relevant evidence	11-13
Generalisability	21	Discuss the generalisability (external validity) of the study results	13
**Other information**			
Funding	22	Give the source of funding and the role of the funders for the present study and, if applicable, for the original study on which the present article is based	18

*Give information separately for cases and controls in case-control studies and, if applicable, for exposed and unexposed groups in cohort and cross-sectional studies.

For peer review only

Note: An Explanation and Elaboration article discusses each checklist item and gives methodological background and published examples of transparent reporting. The STROBE checklist is best used in conjunction with this article (freely available on the Web sites of PLoS Medicine at http://www.plosmedicine.org/, Annals of Internal Medicine at http://www.annals.org/, and Epidemiology at http://www.epidem.com/). Information on the STROBE Initiative is available at www.strobe-statement.org.

For peer review only

BMJ Open

Do the associations of body mass index and waist circumference with back pain change as people age? 32 years of follow-up in a British birth cohort

Journal:	*BMJ Open*
Manuscript ID	bmjopen-2020-039197.R1
Article Type:	Original research
Date Submitted by the Author:	30-Sep-2020
Complete List of Authors:	Muthuri, Stella; UCL, MRC Unit for Lifelong Health and Ageing<br>Cooper, Rachel; Manchester Metropolitan University, Sport and Exercise Sciences<br>Kuh, Diana; UCL, MRC Unit for Lifelong Health and Ageing<br>Hardy, Rebecca; UCL, CLOSER, UCL Institute of Education
**Primary Subject Heading**:	Epidemiology
Secondary Subject Heading:	Public health
Keywords:	Back pain < ORTHOPAEDIC & TRAUMA SURGERY, EPIDEMIOLOGY, PUBLIC HEALTH

SCHOLARONE™
Manuscripts

I, the Submitting Author has the right to grant and does grant on behalf of all authors of the Work (as defined in the below author licence), an exclusive licence and/or a non-exclusive licence for contributions from authors who are: i) UK Crown employees; ii) where BMJ has agreed a CC-BY licence shall apply, and/or iii) in accordance with the terms applicable for US Federal Government officers or employees acting as part of their official duties; on a worldwide, perpetual, irrevocable, royalty-free basis to BMJ Publishing Group Ltd ("BMJ") its licensees and where the relevant Journal is co-owned by BMJ to the co-owners of the Journal, to publish the Work in this journal and any other BMJ products and to exploit all rights, as set out in our licence.

The Submitting Author accepts and understands that any supply made under these terms is made by BMJ to the Submitting Author unless you are acting as an employee on behalf of your employer or a postgraduate student of an affiliated institution which is paying any applicable article publishing charge ("APC") for Open Access articles. Where the Submitting Author wishes to make the Work available on an Open Access basis (and intends to pay the relevant APC), the terms of reuse of such Open Access shall be governed by a Creative Commons licence – details of these licences and which Creative Commons licence will apply to this Work are set out in our licence referred to above.

Other than as permitted in any relevant BMJ Author's Self Archiving Policies, I confirm this Work has not been accepted for publication elsewhere, is not being considered for publication elsewhere and does not duplicate material already published. I confirm all authors consent to publication of this Work and authorise the granting of this licence.

For peer review only

Do the associations of body mass index and waist circumference with back pain change as people age? 32 years of follow-up in a British birth cohort

Stella G Muthuri[1], Rachel Cooper[2], Diana Kuh[1], Rebecca Hardy[3*]

[1] MRC Unit for Lifelong Health and Ageing at UCL, London, UK

[2] Musculoskeletal Science and Sports Medicine Research Centre, Department of Sport and Exercise Sciences, Manchester Metropolitan University, Manchester, UK

[3] CLOSER, UCL Institute of Education, University College London, London, UK

*** Corresponding author:** Rebecca Hardy, CLOSER, UCL Social Research Institute, 55-59 Gordon Square, London, WC1H 0NU, UK

Email: rebecca.hardy@ucl.ac.uk

Word count (main text): 3282

Key words: back pain; obesity; adiposity; birth cohort; ageing

Contributors

SGM and RH had full access to all the data in the study and take full responsibility for the integrity and the accuracy of data analysis. SGM, DK, and RC conceived the idea for this study; SGM, DK, RH and RC contributed to the development of the study objectives; DK, RH and RC acquired the data; SGM and RH analysed the data; SGM and RH drafted the manuscript; all authors contributed to the manuscript's critical revision and provided final approval of the version to be published.

For peer review only - http://bmjopen.bmj.com/site/about/guidelines.xhtml

Abstract

Objectives: To investigate whether cross-sectional and longitudinal associations of body mass index (BMI) and waist circumference (WC) with back pain change with age and extend into later life.

Design: British birth cohort study

Setting: England, Scotland and Wales

Participants: Up to 3426 men and women from the MRC National Survey of Health and Development

Primary outcome measures: Back pain (sciatica, lumbago, or recurring/severe backache all or most of the time) was self-reported during nurse interviews at ages 36, 43, 53 and 60-64 years and in a postal questionnaire using a body manikin at age 68.

Results: Findings from mixed-effects logistic regression models indicated that higher BMI was consistently associated with increased odds of back pain across adulthood. Sex-adjusted odds ratios of back pain per 1 standard deviation increase in BMI were: 1.13 (95% CI 1.01, 1.26), 1.11 (95% CI 1.00, 1.23), 1.17 (95% CI 1.05, 1.30), 1.31 (95% CI 1.15, 1.48) and 1.08 (95% CI 0.95, 1.24) at ages 36, 43, 53, 60-64 and 68-69, respectively. Similar patterns of associations were observed for WC. These associations were maintained when potential confounders, including education, occupational class, height, cigarette smoking status, physical activity and symptoms of anxiety and depression were accounted for. BMI showed stronger associations than WC in models including both measures.

Conclusions: These findings demonstrate that higher BMI is a persistent risk factor for back pain across adulthood. This highlights the potential lifelong consequences on back pain of the rising prevalence of obesity within the population.

For peer review only - http://bmjopen.bmj.com/site/about/guidelines.xhtml

Article summary

Strengths and limitations of this study

- The availability of data on back pain, body mass index (BMI) and waist circumference (WC) prospectively ascertained over 32 years of follow-up in a large representative population-based sample is a key and unique strength of this study.
- This allowed us to formally test whether the cross-sectional and longitudinal relationships of BMI and WC with back pain change with age, extend into later life and are independent of each other so addressing important gaps in our current understanding.
- As back pain was not assessed in exactly the same way at different time points across adulthood, timing of onset of pain was not recorded and severity and chronicity were not distinguished results need to be interpreted with some caution.
- Residual confounding cannot be fully ruled out as a potential explanation of our findings however, the risk of this has been minimised by taking account of a wide range of potential confounders measured prospectively across adulthood.
- Cross-sectional associations between body size and back pain may be explained by reverse causality, but we also investigated longitudinal associations between BMI and back pain and our conclusions remained unchanged.

For peer review only

For peer review only - http://bmjopen.bmj.com/site/about/guidelines.xhtml

Introduction

Back pain is one of the most commonly reported musculoskeletal disorders and was recently ranked as the number one cause of disability in most countries worldwide.[1] As it is a major cause of activity limitation and functional decline in later life[2-8] the fact that its prevalence is projected to increase as population ageing continues is a considerable cause for concern. As a result, there have been calls to intensify research efforts to address the public health challenge of the burden of low back pain.[9]

Obesity, usually indicated by high body mass index (BMI), has been identified as an important risk factor for back pain (typically self-reported during a structured interview or self-completion questionnaire).[10-14] However, the majority of existing studies have examined cross-sectional associations at a single time-point, often in early or mid-adulthood[10, 13-19] and/or are in occupational-based cohorts.[10] There are few studies on obesity and back pain in older populations. This is despite evidence to suggest that the aetiology of back pain may change with age as degenerative back disorders become increasingly common; these disorders may have different risk factors to conditions that precipitate back pain earlier in life. This is coupled with reduced pain-modulatory capacity occurring with advancing age partly explaining increases in persistent and disabling pain conditions among older adults.[20] In one study of older populations (age >50 years) from nine countries, high BMI was associated with increased odds of back pain, assessed during an interview using a simple question on experience of back pain in the last 30 days, in European countries (Poland, Russia, Finland, Spain) and South Africa.[21] However, whether or not the associations found among these older adults were weaker, due to potential changes in the underlying aetiology of back pain, or of similar strength to those that would have been observed in these populations if they had been assessed at younger ages could not be

For peer review only

established. To our knowledge, no study has formally investigated whether the association between BMI and back pain changes with age; thus whether or not obesity remains a suitable target for intervention for the prevention of back pain into later life, alongside the important role of interventions targeting obesity to prevent many other chronic conditions, remains to be established.

Higher central adiposity, as well as higher BMI, has been related to increased risk of back pain[12, 15-19, 22] and may be more indicative of inflammatory processes due to increases in fat mass. Despite this, few studies[22] have compared the strength of associations between BMI and abdominal adiposity or tested whether there is any additional effect of abdominal adiposity over and above BMI. This could provide new aetiological insights to help inform the development of interventions to prevent or alleviate back pain.

The MRC National Survey of Health and Development (NSHD), the oldest of the British birth cohort studies, has assessed BMI, waist circumference (WC) and back pain at multiple time points across adulthood from midlife up to age 69. We therefore address important research gaps by first examining whether the cross-sectional relationships of BMI and WC with back pain change with age, extend into later life and are independent of each other. We then assess the prospective associations of BMI and WC with back pain at the subsequent age to assess the extent to which patterns of association with age may be affected by reverse causality.

Subjects and Methods

The NSHD is a socially stratified sample of 5362 single, legitimate births that occurred in England, Wales and Scotland in one week of March 1946. To date the same participants

For peer review only - http://bmjopen.bmj.com/site/about/guidelines.xhtml

have been assessed on up to 24 occasions between birth and age 69, with participation rates remaining relatively high (i.e. >80% of eligible sample responding) across life.[23-25] All waves of data collection have complied with ethical standards. Ethical approval for the most recent data collection at age 68-69 was obtained from the Queen Square Research Ethics Committee (14/LO/1073) and the Scotland A Research Ethics Committee (14/SS/1009). All methods were carried out in accordance with the relevant guidelines and regulations and written informed consent was obtained.

Patient and public involvement

Over the 74 years of this study, the research has increasingly involved participants, in line with changing norms about conducting cohort studies. Participant involvement includes receiving personal letters from the research team as required, and invitations to participate in birthday celebrations, public engagement activities and focus groups to discuss future data collections. When piloting new questionnaires and assessments, including the most recent of those used in these analyses, patients from general practices and the University College London Hospitals Patient Public Involvement group were recruited and asked to provide feedback which was taken into account when designing the mainstage fieldwork.

Back pain assessment

Back pain was ascertained during five of the main assessments of NSHD participants via structured nurse interviews at ages 36, 43, 53 and 60-64 and in a postal questionnaire at age 68 (see Supplementary Methods for questions used). At all ages except age 68, participants were asked whether they had sciatica, lumbago, or recurring/severe backache all or most of the time (ever at ages 36 and 43 and in the previous 12 months at ages 53 and 60-64). At age 68, participants were asked whether they had experienced any ache or pain in the previous

For peer review only - http://bmjopen.bmj.com/site/about/guidelines.xhtml

month which had lasted for one day or longer, not including pain occurring during the course of a feverish illness such as flu. Those who responded positively were asked to shade the location of their pain using a four-view body manikin. Those who shaded any back site were classified as having back pain for the purposes of these analyses.

Anthropometric measurements

Height, weight and waist circumference (taken at the midpoint between the costal margin and iliac crest) were measured by nurses using standardised protocols at ages 36, 43, 53, 60-64 and 69 years. BMI was calculated at each age as weight (kg)/height (m)^2. BMI and waist circumference at each age were sex-standardised (to a mean of 0 and standard deviation (SD) of 1 (calculated as (x-mean$_i$)/SD$_j$ where x is the raw measure and mean$j$ and SDj are the sample mean and SD for sex j); our units of analysis for BMI and waist circumference are both 1SD to facilitate comparisons of effect sizes across age and sex.

Fat and lean mass at 60-64 years

Measures of body composition were obtained in participants who attended one of six Clinical Research facilities when aged 60-64 years using a QDR 4500 Discovery DXA scanner (Hologic Inc, Bedford, MA, USA) whilst the individuals were in a supine position. Whole body fat mass was measured and whole body lean mass was calculated as total mass minus fat and bone mass. Mass from the head was excluded and measures were converted to kg. Full details are provided elsewhere.[26] Fat mass index (kg/m^2) and lean mass index (kg/m^2) were calculated by dividing the measures of mass by height-squared.

Covariates

Covariates representing different domains of the biopsychosocial model of pain were selected

For peer review only - http://bmjopen.bmj.com/site/about/guidelines.xhtml

a priori.[27] This included variables that have previously been identified as key risk factors for back pain and could potentially confound the main associations.[28-30] These were sex, educational attainment at age 26, occupational class at age 53 and measures of height, smoking status, physical activity, and symptoms of anxiety and depression, which were available at ages 36, 43, 53, 60-64 and 68-69 and so included as time varying covariates.

The highest education level achieved by age 26 was grouped into no qualifications, up to O-level or equivalent, or A-level or equivalent and above. Own occupation at age 53 was categorised according to the Registrar General's social classification into three groups: high (I or II: professional, managerial or technical); middle (IIINM skilled non-manual or IIIM: skilled manual); low (IV or V: partly skilled or unskilled manual).

Smoking status was assessed by self-report at each wave and categorised as never, ex and current smoker. Participation in sports, vigorous leisure activities or exercise was assessed at each wave and participants were grouped as inactive, moderately active (1-4 times/month) or regularly active (≥5 times/month).[31]

Symptoms of anxiety and depression were also assessed at all five waves. At age 36, a shortened version of the Present State Examination[32] was used at a nurse interview and the total score was recoded to a binary variable using the recommended threshold for caseness of 5 or more. At age 43, participants completed the 18-item Psychiatric Symptom Frequency (PSF) scale[33] and the total score was calculated and then dichotomised into absence of symptoms (PSF score <23) and presence of symptoms (PSF score ≥23). At ages 53, 60-64 and 68/69 the 28-item General Health Questionnaire (GHQ-28)[34] was self-administered

For peer review only - http://bmjopen.bmj.com/site/about/guidelines.xhtml

and scores for all items were summed and further dichotomised using a recommended threshold for caseness of 5 or more.

Statistical analysis

We first examined descriptive statistics for each variable and formally tested sex differences in their distributions using chi-squared and t-tests as appropriate.

Multilevel mixed-effects logistic regression models with a random intercept were then used to test associations between BMI and back pain with measurement occasion nested within individuals. These models allow for the correlation between measurements on the same individual. We fitted measurement wave as a categorical variable with age 36 as the reference category and adjusted for sex. Interactions between wave and sex were tested in the model however, as there was no evidence of interactions these were removed in subsequent models. We then added BMI as a time varying covariate. Formal assessment of whether associations between BMI and back pain varied by sex were performed by including sex by BMI interaction terms in models and where no evidence of interaction was found models were sex-adjusted. Deviations from linearity were assessed by including quadratic terms for BMI but no evidence of this was found. To assess whether the association of BMI with back pain changed with age, interactions between wave and BMI were included (Model 1). The model was then adjusted for potential confounding variables (Model 2). Analyses were repeated replacing BMI with WC and then a final confounder adjusted model was run in which BMI and WC were included together. All models included the maximum number of participants, which was those participants with a valid measure of back pain, BMI, WC and all covariates for at least one age (n=3426) (see table S1 for number of participants who contributed data at each wave).

For peer review only - http://bmjopen.bmj.com/site/about/guidelines.xhtml

We then repeated the same modelling approach to assess prospective associations between BMI, WC and back pain. To achieve this, we used back pain at 43, 53, 60-64 and 68 as outcomes and related them to BMI and WC at the prior age (i.e. at ages 36, 43, 53 and 60-64). These models included a sample size of 3044.

Finally, in the sub-sample with body composition measures at age 60-64 (n=1186) supplementary analyses using multiple regression models, assessed the associations between fat mass index and lean mass index at 60-64 years and back pain at 68 years. Model 1 included sex and both body composition measures and model 2 added the same covariates used in previous models.

All analyses were performed using STATA version 14.1.

Results

The characteristics of the study sample are shown in Table 1 (and Table S2). Overall, the percentage reporting back pain varied from 17.9% and 32.3% and was higher in women than men at every age (Table 1).

There was no evidence of an interaction between sex and either BMI (p=0.2) or waist circumference (p=0.9) and so the main models were sex-adjusted. In sex-adjusted models, higher BMI was associated with increased odds of back pain at every age (Table 2). Sex-adjusted odds ratios (ORs) of back pain per 1SD increase in BMI estimated from this model were 1.13 (95% CI 1.01, 1.26), 1.11 (95% CI 1.00, 1.23), 1.17 (95% CI 1.05, 1.30), 1.31 (95% CI 1.15, 1.48) and 1.08 (95% CI 0.95, 1.24) at ages 36, 43, 53, 60-64 and 68-69,

For peer review only - http://bmjopen.bmj.com/site/about/guidelines.xhtml

respectively. There was no evidence of a difference in association between age 36 and ages 43, 53, or 68 (wave by BMI interactions p>0.5) and only weak evidence that the association at age 60-64 was stronger than at age 36 (age 60-64 wave by BMI interaction: p=0.07) (Supplementary Table S3). Adjustment for potential confounders had minimal impact on these findings and estimated ORs for each age remained similar (Table 2).

A similar, and slightly less variable, pattern of association was observed for WC, with no evidence that the association at age 60-64 was stronger than at other ages (wave by BMI interaction: p=0.2) (Table 2, Supplementary Table S3). Adjustment for potential confounders attenuated all ORs somewhat, and to a greater extent than the ORs for BMI (Table 2).

Including both BMI and WC in the same fully adjusted model with all age interaction terms led to inflated standard errors. We therefore included only the age 60-64 wave by BMI and the age 60-64 wave by WC interaction, finding stronger evidence of the interaction with BMI. Our final fully adjusted model thus included only the interaction between BMI and age 60-64; the association of BMI with back pain (OR per SD (95% CI): 1.11 (0.99, 1.24) at 36, 43, 53 and 68-69y and 1.28 (1.09,1.50) at 60-64y) was stronger than that for WC (OR per SD (95% CI): 1.02 (0.91,1.13)) at all ages.

Findings for both BMI and WC were very similar in the longitudinal analysis, when relating the body size measure at the prior age to subsequent back pain (Table 3). In supplementary analyses (n=1186), higher fat mass index was associated with higher odds of back pain at age 68 (OR per SD (95% CI): 1.23 (1.04, 1.45)) in a model adjusting for sex and lean mass index, but no association was observed for lean mass index (Supplementary Table S4). The

For peer review only - http://bmjopen.bmj.com/site/about/guidelines.xhtml

association with fat mass index was maintained after adjustment for other potential confounders.

Discussion

In a large nationally representative British birth cohort higher BMI and WC were consistently associated with increased odds of back pain between ages 36 and 68. These associations were maintained after adjustment for confounders and, BMI remained more strongly related to back pain than WC in a model including both variables. Findings were similar when prospective associations were investigated for back pain between 43 and 68.

Our findings are consistent with previous studies which have reported associations between overweight and obesity, indicated by high BMI, and increased odds of back pain.[10] Findings from cross-sectional studies also suggest positive associations between higher WC and low back pain in women, but not in men.[12, 15-19] These previous studies have sampled participants across a range of different ages (15 years and older) and although they have adjusted for age, none has examined whether associations of BMI and WC with back pain vary with age or whether associations remain into older age. Our study shows persisting associations of BMI and WC with back pain until age 68 years with little evidence of variation with age.

Another key finding is that BMI exhibited a stronger association than WC in a model including both measures. This suggests that mechanisms specifically related to central adiposity do not fully underlie the associations observed. This contrasts with a previous study in young adults (age 24-39 years) which found that WC but not BMI remained associated with back pain in mutually adjusted models in women only.[15] Our findings in relation to fat

For peer review only - http://bmjopen.bmj.com/site/about/guidelines.xhtml

and lean mass suggest that associations with BMI may be driven by whole body fat mass, rather than abdominal fat mass specifically. In testing these associations we are addressing the call made in a recent systematic review for further high-quality studies on the relationships of body composition with pain.[35] Our findings are consistent with a prospective study[12] included in this review that found evidence that fat mass was associated with low back pain intensity and disability in both sexes. Likewise, a cross-sectional study found that higher fat mass (independent of lean mass) was associated with higher levels of low back pain intensity and disability but found no relationship with lean mass after adjusting for fat mass.[11] The stronger associations with BMI than WC may point to the importance of mechanical effects, with increased BMI resulting in higher compressive force on the spine during activity.[10] However, we cannot rule out the fact that chronic inflammatory or metabolic pathways may also be relevant. For example, atherosclerosis and chronic inflammation, which are linked to obesity, have also been associated with disc degeneration and low back pain.[36, 37]

A key strength of our study is the large representative population-based sample of adults with assessments of back pain and measured height, weight and waist circumference at five time points over 32 years of follow-up. We assessed back pain using simple questions asked during structured nurse interviews and in a self-completed questionnaire which are commonly used methods in population-based studies of back pain.[10-14, 21] The prevalence estimates of back pain in our study at different ages were comparable to those reported in other studies,[2] and we were able to show that associations with BMI are consistent across adulthood and persist into old age. Another strength is the availability of data on a wide range of potential confounders measured prospectively across adulthood that allowed us to adjust for a range of time-varying covariates and reduce the risk of residual confounding.

For peer review only - http://bmjopen.bmj.com/site/about/guidelines.xhtml

Alongside key strengths, our results need interpreting in the context of a number of potential limitations. Firstly, while our study population were selected to be nationally representative at birth and due to high participation rates across life have remained so in many respects,[23-25] bias may have been introduced due to the necessary exclusion of all those lost to follow-up before age 36 and/or with missing data on back pain, body size and covariates at all 5 waves. Secondly, cross-sectional associations observed between body size and back pain may be at least partly explained by reverse causality, especially at the latest wave when back pain was measured at age 68 years, a year prior to the assessment of body size due to the design of the data collection at age 68-69 years. However, we also investigated associations between back pain and BMI measured at a prior age and findings remained unchanged. A third potential limitation, relating to the fact that our analyses are post-hoc i.e. data are drawn from a large population-based study designed to capture information on a wide range of different measures of health and their risk factors across life rather than to address this specific research question, is that back pain was not assessed in exactly the same way at different time points across adulthood. In addition, it was not possible to establish the severity and chronicity of back pain, and the validity of the back pain assessments used has not been evaluated. Finally, our study population comprised Caucasian men and women born in Britain in 1946. While this allows us to rule out confounding by age or ethnicity as potential explanations of our findings, it may limit their generalisability. Further research is thus necessary to consider the implications of these findings for more ethnically diverse cohorts and also more recently born cohorts, the latter who are likely to have spent more of their lives overweight or obese.[38]

In summary, our findings suggest that there is a consistent relationship between higher

For peer review only - http://bmjopen.bmj.com/site/about/guidelines.xhtml

adiposity and back pain across adulthood. Our results also show that WC does not add further information once BMI has been accounted for in this relationship. This underscores the importance of primary and secondary interventions to prevent excessive weight gain and reduce overweight and obesity across the whole of adulthood in order to help prevent back pain and its disabling consequences as individuals age.

For peer review only

For peer review only - http://bmjopen.bmj.com/site/about/guidelines.xhtml

Acknowledgements

The authors are grateful to NSHD study members for their continuing participation in the study. We also thank members of the NSHD scientific and data collection teams.

Funding statement

This work was supported by the UK Medical Research Council (Programme codes: MC_UU_12019/4 and MC_UU_12019/2). RH is Director of the CLOSER consortium which is supported by funding from the Economic and Social Research Council (ESRC) (award reference: ES/K000357/1).

For peer review only

Competing interests

The authors declare no financial or non-financial competing interests

Data sharing statement

Data used in this publication are available to bona fide researchers upon request to the NSHD Data Sharing Committee via a standard application procedure. Further details can be found at http://www.nshd.mrc.ac.uk/data. doi: 10.5522/NSHD/Q101; doi: 10.5522/NSHD/Q102; 10.5522/NSHD/Q103

For peer review only - http://bmjopen.bmj.com/site/about/guidelines.xhtml

References

1. GBD 2015 Disease and Injury Incidence and Prevalence Collaborators. Global, regional, and national incidence, prevalence, and years lived with disability for 310 diseases and injuries, 1990-2015: a systematic analysis for the Global Burden of Disease Study 2015. *Lancet* 2016;388:1545-602.

2. Dionne CE, Dunn KM, Croft PR. Does back pain prevalence really decrease with increasing age? A systematic review. *Age Ageing* 2006;35:229-34.

3. Docking RE, Fleming J, Brayne C, et al. Epidemiology of back pain in older adults: prevalence and risk factors for back pain onset. *Rheumatology* 2011;50:1645-53.

4. Leveille SG, Bean J, Ngo L, et al. The pathway from musculoskeletal pain to mobility difficulty in older disabled women. *Pain* 2007;128:69-77.

5. Patel KV, Guralnik JM, Dansie EJ, et al. Prevalence and impact of pain among older adults in the United States: findings from the 2011 National Health and Aging Trends Study. *Pain* 2013;154:2649-57.

6. Marshall LM, Litwack-Harrison S, Cawthon PM, et al. A prospective study of back pain and risk of falls among older community-dwelling women. *J Gerontol Med Sci* 2016;71:1177-83.

7. O'Sullivan K, Kennedy N, Purtill H, et al. Understanding pain among older persons: Part 1-the development of novel pain profiles and their association with disability and quality of life. *Age Ageing* 2017;46:46-51.

8. Kennedy N, O'Sullivan K, Hannigan A, et al. Understanding pain among older persons: Part 2—the association between pain profiles and healthcare utilisation. *Age Ageing* 2017;46:51-6.

9. Hartvigsen J, Hancock MJ, Kongsted A, et al. What low back pain is and why we need to pay attention. *Lancet* 2018;391:2356-67.

For peer review only - http://bmjopen.bmj.com/site/about/guidelines.xhtml

10. Shiri R, Karppinen J, Leino-Arjas P, et al. The association between obesity and low back pain: A meta-analysis. *Am J Epidemiol* 2010;171:135-54.

11. Urquhart DM, Berry P, Wluka AE, et al. 2011 Young Investigator Award winner: Increased fat mass is associated with high levels of low back pain intensity and disability. *Spine (Phila Pa 1976)* 2011;36:1320-5.

12. Hussain SM, Urquhart DM, Wang Y, et al. Fat mass and fat distribution are associated with low back pain intensity and disability: results from a cohort study. *Arthritis Res Ther* 2017;19:26.

13. Heuch I, Hagen K, Heuch I, et al. The impact of body mass index on the prevalence of low back pain: the HUNT study. *Spine (Phila Pa 1976)* 2010;35:764-8.

14. Heuch I, Heuch I, Hagen K, et al. Body mass index as a risk factor for developing chronic low back pain: a follow-up in the Nord-Trondelag Health Study. *Spine (Phila Pa 1976)* 2013;38:133-9.

15. Shiri R, Solovieva S, Husgafvel-Pursiainen K, et al. The association between obesity and the prevalence of low back pain in young adults. The Cardiovascular Risk in Young Finns Study. *Am J Epidemiol* 2008;167:1110-9.

16. Han TS, Schouten JS, Lean ME, et al. The prevalence of low back pain and associations with body fatness, fat distribution and height. *Int J Obes Relat Metab Disord* 1997;21:600-97.

17. Toda Y, Segal N, Toda T, et al. Lean body mass and body fat distribution in participants with chronic low back pain. *Arch Intern Med* 2000;160:3265-9.

18. Lean ME, Han TS, Seidell JC. Impairment of health and quality of life in people with large waist circumference. *Lancet* 1998;351:853-6.

19. Frilander H, Solovieva S, Mutanen P, et al. Role of overweight and obesity in low back disorders among men: a longitudinal study with a life course approach. *BMJ Open*

For peer review only - http://bmjopen.bmj.com/site/about/guidelines.xhtml

2015;5:e007805.

20. Gagliese L. Pain and aging: The emergence of a new subfield of pain research. *J Pain* 2009;10:343-53.

21. Koyanagi A, Stickley A, Garin N, et al. The association between obesity and back pain in nine countries: a cross-sectional study. *BMC Public Health* 2015;15:123.

22. Heuch I, Heuch I, Hagen K, et al. A comparison of anthropometric measures for assessing the association between body size and risk of chronic low back pain: The HUNT Study. *PLoS One* 2015;10:e0141268.

23. Kuh D, Pierce M, Adams J, et al. Cohort profile: updating the cohort profile for the MRC National Survey of Health and Development: a new clinic-based data collection for ageing research. *Int J Epidemiol* 2011;40:e1-9.

24. Kuh D, Wong A, Shah I, et al. The MRC National Survey of Health and Development reaches age 70: maintaining participation at older ages in a birth cohort study. *Eur J Epidemiol* 2016;31:1135-47.

25. Wadsworth M, Kuh D, Richards M, et al. Cohort Profile: The 1946 National Birth Cohort (MRC National Survey of Health and Development). *Int J Epidemiol* 2006;35:49-54.

26. Bann D, Kuh D, Wills AK, et al. Physical activity across adulthood in relation to fat and lean body mass in early old age: findings from the Medical Research Council National Survey of Health and Development, 1946-2010. *Am J Epidemiol* 2014;179:1197-207.

27. Gatchel RJ, Peng YB, Peters ML, et al. The biopsychosocial approach to chronic pain: Scientific advances and future directions. *Psychol Bull* 2007;133:581-624.

28. Hoy D, Brooks P, Blyth F, et al. The epidemiology of low back pain. *Best Pract Res Clin Rheumatol* 2010;24:769-81.

29. Heuch I, Heuch I, Hagen K, et al. Association between body height and chronic low back pain: a follow-up in the Nord-Trøndelag Health Study. *BMJ Open* 2015;5:e006983.

For peer review only - http://bmjopen.bmj.com/site/about/guidelines.xhtml

30. Kherad M, Rosengren BE, Hasserius R, et al. Risk factors for low back pain and sciatica in elderly men-the MrOS Sweden study. *Age Ageing* 2017;46:64-71.

31. Elhakeem A, Cooper R, Bann D, et al. Birth weight, school sports ability, and adulthood leisure-time physical activity. *Med Sci Sports Exerc* 2017;49:64-70.

32. Wing J, Cooper J, Sartorius N. The measurement and classification of psychiatric symptoms. Cambridge: Cambridge University Press; 1974.

33. Lindelow M, Hardy R, Rodgers B. Development of a scale to measure symptoms of anxiety and depression in the general UK population: the psychiatric symptom frequency scale. *J Epidemiol Community Health* 1997;51:549-57.

34. Goldberg DP, Hillier VF. A scaled version of the General Health Questionnaire. *Psychol Med* 1979;9:139-45.

35. Walsh TP, Arnold JB, Evans AM, et al. The association between body fat and musculoskeletal pain: a systematic review and meta-analysis. *BMC Musculoskelet Disord* 2018;19:233.

36. Kauppila LI. Atherosclerosis and disc degeneration/low-back pain-a systematic review. *Eur J Vasc Endovasc Surg* 2009;37:661-70.

37. Ruiz-Fernández C, Francisco V, Pino J, et al. Molecular relationships among obesity, inflammation and intervertebral disc degeneration: Are adipokines the common link? *Int J Mol Sci* 2019;20:E2030.

38. Johnson W, Li L, Kuh D, et al. How has the age-related process of overweight or obesity development changed over time? Co-ordinated analyses of individual participant data from five United Kingdom birth cohorts. *PLoS Med* 2015;12:e1001828.

For peer review only - http://bmjopen.bmj.com/site/about/guidelines.xhtml

Table 1: Characteristics of the MRC National Survey of Health and Development sample included in analysis [a]

		Male	Female	p-value[b]
**Back pain, N (%), at age:**				
36y				.08
	No	1283 (83.3)	1256 (80.9)	
	Yes	257 (16.7)	297 (19.1)	
43y				.06
	No	1106 (74.6)	1042 (71.5)	
	Yes	377 (25.4)	415 (28.6)	
53y				.06
	No	925 (69.4)	903 (66.0)	
	Yes	407 (30.6)	465 (34.0)	
60-64y				.06
	No	629 (72.1)	643 (68.1)	
	Yes	243 (27.9)	301 (31.9)	
68y				.02
	No	637 (74.1)	635 (69.2)	
	Yes	223 (25.9)	282 (30.8)	
**BMI (kg/m^2), mean (SD), at age:**				
36y		24.8 (3.3)	23.5 (4.1)	<.001
43y		25.7 (3.5)	25.1 (4.7)	<.001
53y		27.4 (4.1)	27.4 (5.4)	.85
60-64y		27.9 (4.1)	27.9 (5.5)	.99
69y		28.1 (4.5)	28.0 (5.6)	.93
**Waist circumference (cm), mean (SD), at age:**				
36y		89.5 (9.3)	76.9 (11.5)	<.001
43y		91.8 (9.9)	77.7 (11.2)	<.001
53y		97.7 (10.9)	85.8 (12.8)	<.001
60-64y		100.8 (11.1)	92.2 (13.0)	<.001
69y		100.9 (12.3)	92.1 (14.0)	<.001

Note. BMI: body mass index; WC: waist circumference; SD: Standard Deviation; y: years

[a] Maximum N=3426 (1723 males and 1703 females) (this includes participants with a valid measure of back pain, BMI, waist circumference and each covariate for at least one age). Number of participants contributing data at each age: 36y, N=3093; 43y, N=2940; 53y, N=2700; 60-64y, N=1816; 68-69y, N=1777 (see Supplementary table S1 for more details)

[b] p-values from formal tests of sex differences using chi-squared and t-tests as appropriate

For peer review only - http://bmjopen.bmj.com/site/about/guidelines.xhtml

Table 2: Odds Ratios (OR) of back pain at each age per 1 standard deviation increases in BMI and waist circumference at the same age estimated from multilevel logistic models (12 326 observations nested within 3426 individuals)

	BMI			
	Model 1[a]		Model 2[b]	
Per SD BMI at:	OR (95% CI)	P value	OR (95% CI)	P value
36y	1.13 (1.01 , 1.26)	0.03	1.12 (1.00,1.25)	0.05
43y	1.11 (1.00 , 1.23)	0.06	1.11 (1.00,1.23)	0.05
53y	1.17 (1.05 , 1.30)	0.003	1.17 (1.05,1.30)	0.003
60-64y	1.31 (1.15 , 1.48)	<0.001	1.30 (1.14,1.47)	<0.001
68y	1.08 (0.95 , 1.24)	0.2	1.07 (0.94,1.22)	0.3
	Waist circumference			
	Model 1[a]		Model 2[b]	
Per SD WC at:	OR (95% CI)	P value	OR (95% CI)	P value
36y	1.17 (1.05 , 1.31)	0.005	1.14 (1.02,1.27)	0.02
43y	1.12 (1.01 , 1.24)	0.03	1.08 (0.98,1.20)	0.1
53y	1.16 (1.05 , 1.29)	0.005	1.13 (1.02,1.25)	0.03
60-64y	1.27 (1.12 , 1.44)	<0.001	1.21 (1.07,1.38)	0.003
68y	1.12 (0.98 , 1.27)	0.1	1.07 (0.94,1.22)	0.3

Note. BMI: body mass index; SD: Standard Deviation; OR: Odds Ratio; y: years

Please see Table S5 for results from analyses rerun with BMI and WC modelled in raw units, i.e. kg/m^2 and cm, respectively

[a] Model 1: Includes age as a categorical variable, standardised BMI or WC (mean=0, SD=1) and an age by BMI or age by WC interaction (as appropriate), adjusted for sex (as there was no evidence of a sex by BMI (p=0.2) or a sex by WC (p=0.9) interaction)

For peer review only - http://bmjopen.bmj.com/site/about/guidelines.xhtml

[b] Model 2: Includes age as a categorical variable, standardised BMI or WC (mean=0, SD=1) and an age by BMI or age by WC interaction (as appropriate), adjusted for sex, education, occupational class and time-varying covariates (height, cigarette smoking status, physical activity and symptoms of anxiety and depression)

For peer review only

For peer review only - http://bmjopen.bmj.com/site/about/guidelines.xhtml

Table 3: Odds Ratios (OR) of back pain at each age per 1 standard deviation increase in BMI and waist circumference at the previous age estimated from multilevel logistic models (8 595 observations nested within 3044 individuals)

		BMI			
		Model 1[a]		Model 2[b]	
Per SD BMI/WC at age:	Back pain at age:	OR (95% CI)	P value	OR (95% CI)	P value
36y	43y	1.08 (0.97 , 1.20)	0.2	1.06 (0.96,1.18)	0.3
43y	53y	1.17 (1.05 , 1.31)	0.004	1.16 (1.04,1.29)	0.009
53y	60-64y	1.32 (1.15 , 1.51)	<0.001	1.29 (1.13,1.48)	<0.001
60-64y	68y	1.11 (0.96 , 1.28)	0.2	1.08 (0.94,1.25)	0.3
		Waist circumference			
		Model 1[a]		Model 2[b]	
		OR (95% CI)	P value	OR (95% CI)	P value
36y	43y	1.12 (1.01 , 1.24)	0.04	1.08 (0.97,1.21)	0.1
43y	53y	1.17 (1.05 , 1.30)	0.004	1.14 (1.02,1.26)	0.02
53y	60-64y	1.28 (1.12 , 1.47)	<0.001	1.23 (1.07,1.40)	0.003
60-64y	68y	1.10 (0.96 , 1.26)	0.2	1.06 (0.92,1.21)	0.4

Note. BMI: body mass index; SD: Standard Deviation; OR: Odds Ratio; y: years

Please see Table S6 for results from analyses rerun with BMI and WC modelled in raw units, i.e. kg/m^2 and cm, respectively

[a] Model 1: Includes age as a categorical variable, standardised BMI or WC (mean=0, SD=1) and an age by BMI or age by WC interaction (as appropriate), adjusted for sex (as there was no evidence of a sex by BMI (p=0.5) or a sex by WC (p=0.9) interaction)

For peer review only - http://bmjopen.bmj.com/site/about/guidelines.xhtml

[b] Model 2: Includes age as a categorical variable, standardised BMI or WC (mean=0, SD=1) and an age by BMI or age by WC interaction (as appropriate), adjusted for sex, education, occupational class and time-varying covariates (height, cigarette smoking status, physical activity and symptoms of anxiety and depression)

For peer review only

For peer review only - http://bmjopen.bmj.com/site/about/guidelines.xhtml

Supplementary information: Do the associations of body mass index and waist circumference with back pain change as people age? 32 years of follow-up in a British birth cohort

Stella G Muthuri, Rachel Cooper, Diana Kuh & Rebecca Hardy

For peer review only

For peer review only - http://bmjopen.bmj.com/site/about/guidelines.xhtml

Supplementary methods:

Questions used to assess back pain in the MRC National Survey of Health and Development

Ages 36 and 43 years [nurse interviews]:

Do you have any of the following (sciatica, lumbago or recurring backache) all or most of the time?

0 No

1 Yes

Ages 53 and 60-64 years [nurse interviews]:

In the last 12 months, have you had sciatica, lumbago or severe backache?

0 No

1 Yes

Age 68 years [self-completion questionnaire]:

In the *last month,* have you had any ache or pain which has lasted for *one day or longer*? (Please do not include pain occurring only during the course of a feverish illness such as flu)

0 No

1 Yes

Below you will find four diagrams of the body.

Please **shade** in all the places where you have felt or feel the aches and pains.

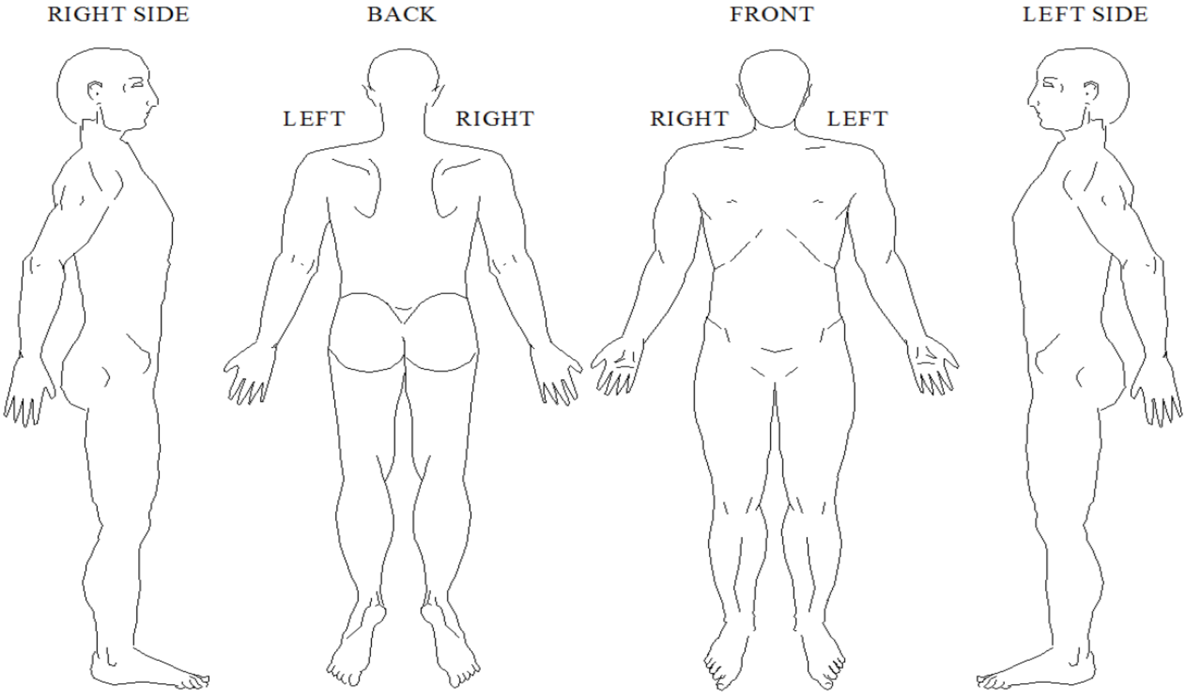

For peer review only - http://bmjopen.bmj.com/site/about/guidelines.xhtml

Table S1: Number of MRC National Survey of Health and Development participants contributing data at each age and number with missing data[a]

Age	Total N contributing data at specified age (no. of males; no. of females)	N with data on back pain but missing data on BMI/WC at specified age	N with data on back pain and BMI/WC at specified age but missing data on covariates
36y	3093 (1540; 1553)	48	175
43y	2940 (1483; 1457)	52	259
53y	2700 (1332; 1368)	44	243
60-64y	1816 (872; 944)	29	390
68-69y	1777 (860; 917)	485	158

[a] A total of 3426 MRC NSHD participants were included in the main analyses. Participant's data could be included at a specified age if there was complete information on back pain, BMI, waist circumference and all covariates at that age
BMI: Body mass index; WC: waist circumference

For peer review only

Table S2: Characteristics of the MRC National Survey of Health and Development sample included in analysis [a]

	Male	Female
**Highest education level achieved by age 26y, N (%)**		
A-level/equivalent or above	706 (41.0)	463 (27.2)
O-level/equivalent or below	357 (20.7)	587 (34.5)
None	660 (38.3)	653 (38.3)
**Own occupational class at age 53y, N (%)**		
High (I/II)	853 (49.5)	593 (34.8)
Middle (IIINM/IIIM)	662 (38.4)	740 (43.5)
Low (IV/V)	208 (12.1)	370 (21.7)
**Time varying covariates[b]**		
**Height (cm), mean (SD), at age:**		
36y	175.4 (6.5)	162.4 (6.0)
43y	175.2 (6.6)	162.4 (6.2)
53y	174.7 (6.5)	161.6 (5.9)
60-64y	174.8 (6.5)	161.9 (5.9)
69y	174.0 (6.4)	160.7 (5.9)
**Smoking status, N (%), at age:**		
36y		
Current	530 (34.4)	524 (33.7)
Ex	617 (40.1)	518 (33.4)
Never	393 (25.5)	511 (32.9)
43y		
Current	460 (31.0)	417 (28.6)
Ex	522 (35.2)	373 (25.6)
Never	501 (33.8)	667 (45.8)
53y		
Current	323 (24.3)	315 (23.0)
Ex	544 (40.8)	391 (28.6)
Never	465 (34.9)	662 (48.4)
60-64y		
Current	97 (11.1)	105 (11.1)
Ex	410 (47.0)	322 (34.1)
Never	365 (41.9)	517 (54.8)
68-69y		
Current	76 (8.8)	65 (7.1)
Ex	411 (47.8)	314 (34.2)
Never	373 (43.4)	538 (58.7)
**Leisure time physical activity, N (%), at age:**		
36y		
Inactive	475 (30.8)	651 (41.9)
Less active	407 (26.4)	376 (24.2)
Most active	658 (42.7)	526 (33.9)
43y		

For peer review only

For peer review only - http://bmjopen.bmj.com/site/about/guidelines.xhtml

	Inactive	714 (48.2)	805 (55.3)
	Less active	354 (23.9)	338 (23.2)
	Most active	415 (28.0)	314 (21.6)
53y			
	Inactive	627 (47.1)	689 (50.4)
	Less active	252 (18.9)	227 (16.6)
	Most active	453 (34.0)	452 (33.0)
60-64y			
	Inactive	557 (63.9)	585 (62.0)
	Less active	118 (13.5)	142 (15.0)
	Most active	197 (22.6)	217 (23.0)
68-69y			
	Inactive	491 (57.1)	520 (56.7)
	Less active	101 (11.7)	141 (15.4)
	Most active	268 (31.2)	256 (27.9)
**Symptoms of anxiety and depression, N(%), at age:**			
36y			
	No	1482 (96.2)	1420 (91.4)
	Yes	58 (3.8)	133 (8.6)
43y			
	No	1350 (91.0)	1233 (84.6)
	Yes	133 (9.0)	224 (15.4)
53y			
	No	1147 (86.1)	1035 (75.7)
	Yes	185 (13.9)	333 (24.3)
60-64y			
	No	765 (87.7)	739 (78.3)
	Yes	107 (12.3)	205 (21.7)
69y			
	No	781 (90.8)	764 (83.3)
	Yes	79 (9.2)	153 (16.7)

SD: Standard Deviation; y: years

[a] Maximum N=3426 (this includes participants with a valid measure of back pain, BMI, waist circumference and each covariate for at least one age)

[b] See number of participants with valid data at specified age in Supplementary Table S1

For peer review only

Table S3: Coefficients from the multilevel models for standardised BMI and waist circumference with back pain across adulthood (N=3426)

	BMI			
	Model 1		Model 2[a]	
	Log-odds (SE)	P value	Log-odds (SE)	P value
BMI (per SD)	0.119 (0.071)	0.03	0.112 (0.056)	0.05
Sex (female)	0.221 (0.072)	0.002	0.500 (0.102)	<0.001
Age 43	0.683 (0.073)	<0.001	0.674 (0.074)	<0.001
Age 53	1.008 (0.074)	<0.001	0.993 (0.076)	<0.001
Age 60-64	0.869 (0.083)	<0.001	0.895 (0.087)	<0.001
Age 68	0.786 (0.084)	<0.001	0.876 (0.088)	<0.001
Age 43*BMI	-0.019 (0.071)	0.8	-0.009 (0.071)	0.9
Age 53*BMI	0.037 (0.072)	0.5	0.045 (0.072)	0.5
Age 60-64*BMI	0.148 (0.082)	0.07	0.148 (0.081)	0.07
Age 68*BMI	-0.040 (0.084)	0.6	-0.046 (0.084)	0.6

	Waist circumference			
	Model 1		Model 2[a]	
	Log-odds (SE)	P value	Log-odds (SE)	P value
WC (per SD)	0.160 (0.057)	0.005	0.129 (0.057)	0.02
Sex (female)	0.221 (0.071)	0.002	0.442 (0.102)	<0.001
Age 43	0.684 (0.073)	<0.001	0.674 (0.074)	<0.001
Age 53	1.010 (0.074)	<0.001	0.990 (0.076)	<0.001
Age 60-64	0.875 (0.083)	<0.001	0.896 (0.087)	<0.001
Age 68	0.787 (0.084)	<0.001	0.867 (0.088)	<0.001
Age 43*WC	-0.048 (0.072)	0.5	-0.049 (0.072)	0.5
Age 53*WC	-0.011 (0.073)	0.9	-0.009 (0.073)	0.9
Age 60-64*WC	0.076 (0.083)	0.4	0.063 (0.083)	0.5
Age 68*WC	-0.050 (0.084)	0.6	-0.058 (0.084)	0.5

Note. BMI: body mass index; WC: waist circumference; SE: standard error; SD: Standard Deviation

As BMI and WC are standardised and so have a mean of 0 and SD of 1, the main effects of age represent the estimate at the mean BMI

[a] Model 2: model 1 + education, occupational class and time-varying covariates (height, cigarette smoking status, physical activity and symptoms of anxiety and depression)

For peer review only - http://bmjopen.bmj.com/site/about/guidelines.xhtml

Table S4: Associations between body composition measures[a] at age 60-64 and back pain at age 68 (n=1186)

Sex-standardised body composition measures	Model 1[b] Odds ratio (95%CI)	Model 1[b] P value	Model 2[c] Odds ratio (95%CI)	Model 2[c] P value
Lean mass index	0.93 (0.79, 1.10)	0.4	0.91 (0.77, 1.08)	0.3
Fat mass index	1.23 (1.04, 1.45)	0.01	1.24 (1.04, 1.47)	0.02

[a]Assessment of body composition measures has been described in detail by Bann et al[1]

[b] Model 1: includes sex, lean mass index and fat mass index

[c] Model 2: model 1 + education at age 26, occupational class at age 53 and the following covariates (assessed at age 60-64): height, cigarette smoking status, physical activity and symptoms of anxiety and depression

For peer review only

Table S5: Odds Ratios (OR) of back pain at each age per 1 kg/m^2 increases in BMI and 1 cm increases in waist circumference at the same age estimated from multilevel logistic models (12 326 observations nested within 3426 individuals)

	BMI			
	Model 1[a]		Model 2[b]	
Per kg/m^2 BMI at:	OR (95% CI)	P value	OR (95% CI)	P value
36y	1.03 (1.00,1.07)	0.04	1.03 (1.00,1.06)	0.05
43y	1.02 (1.00,1.05)	0.06	1.03 (1.00,1.05)	0.05
53y	1.04 (1.01,1.06)	0.002	1.04 (1.01,1.06)	0.002
60-64y	1.06 (1.03,1.08)	<0.001	1.05 (1.03,1.08)	<0.001
68y	1.02 (0.99,1.04)	0.2	1.02 (0.99,1.04)	0.2
	Waist circumference			
	Model 1[a]		Model 2[b]	
Per cm WC at:	OR (95% CI)	P value	OR (95% CI)	P value
36y	1.014 (1.004,1.023)	0.005	1.011 (1.001,1.020)	0.03
43y	1.011 (1.002,1.020)	0.01	1.008 (0.999,1.017)	0.07
53y	1.013 (1.005,1,021)	0.002	1.011 (1.002,1,019)	0.01
60-64y	1.019 (1.009,1.030)	<0.001	1.016 (1.006,1.026)	0.002
68y	1.009 (0.999,1.019)	0.07	1.006 (0.996,1.015)	0.3

Note. BMI: body mass index; OR: Odds Ratio; y: years

[a] Model 1: Includes age as a categorical variable, BMI or WC and an age by BMI or age by WC interaction (as appropriate), adjusted for sex (as there was no evidence of a sex by BMI (p=0.5) or sex by WC (p=0.7) interaction)

[b] Model 2: Includes age as a categorical variable, BMI or WC and an age by BMI or age by WC interaction (as appropriate), adjusted for sex, education, occupational class and time-varying covariates (height, cigarette smoking status, physical activity and symptoms of anxiety and depression)

For peer review only - http://bmjopen.bmj.com/site/about/guidelines.xhtml

Table S6: Odds Ratios (OR) of back pain at each age per 1 kg/m^2 increase in BMI and 1 cm increase in waist circumference at the previous age estimated from multilevel logistic models (8 595 observations nested within 3044 individuals)

		BMI			
		Model 1[a]		Model 2[b]	
Per kg/m^2 BMI at age:	Back pain at age:	OR (95% CI)	P value	OR (95% CI)	P value
36y	43y	1.02 (0.99, 1.05)	0.1	1.02 (0.99, 1.05)	0.2
43y	53y	1.04 (1.02, 1.07)	0.002	1.04 (1.02, 1.07)	0.004
53y	60-64y	1.06 (1.03, 1.09)	<0.001	1.05 (1.02, 1.08)	<0.001
60-64y	68y	1.02 (0.99, 1.05)	0.2	1.02 (0.99, 1.05)	0.3
		Waist circumference			
		Model 1[a]		Model 2[b]	
Per cm WC at age:	Back pain at age:	OR (95% CI)	P value	OR (95% CI)	P value
36y	43y	1.012 (1.002,1.021)	0.01	1.009 (0.999, 1.018)	0.07
43y	53y	1.015 (1.006, 1.025)	0.001	1.013 (1.004, 1.022)	0.005
53y	60-64y	1.019 (1.009, 1.030)	<0.001	1.016 (1.005, 1.026)	0.003
60-64y	68y	1.007 (0.996, 1.019)	0.2	1.004 (0.993, 1.015)	0.5

Note. BMI: body mass index; SD: Standard Deviation; OR: Odds Ratio; y: years

[a] Model 1: Includes age as a categorical variable, BMI or WC and an age by BMI or age by WC interaction (as appropriate), adjusted for sex (as there was no evidence of a sex by BMI (p=0.7) or sex by WC (p=0.9) interaction)

[b] Model 2: Includes age as a categorical variable, BMI or WC and an age by BMI or age by WC interaction (as appropriate), adjusted for sex, education, occupational class and time-varying covariates (height, cigarette smoking status, physical activity and symptoms of anxiety and depression)

For peer review only - http://bmjopen.bmj.com/site/about/guidelines.xhtml

References

1. Bann D, Kuh D, Wills AK, et al. Physical activity across adulthood in relation to fat and lean body mass in early old age: Findings from the Medical Research Council National Survey of Health and Development, 1946–2010. *Am J Epidemiol.* 2014;179:1197-207.

For peer review only

For peer review only - http://bmjopen.bmj.com/site/about/guidelines.xhtml

STROBE 2007 (v4) Statement—Checklist of items that should be included in reports of *cohort studies*
Checklist completed for: Do the associations of body mass index and waist circumference with back pain change as people age? 32 years of follow-up in a British birth cohort

Section/Topic	Item #	Recommendation	Reported on page #
**Title and abstract**	1	(*a*) Indicate the study's design with a commonly used term in the title or the abstract	1; 2
		(*b*) Provide in the abstract an informative and balanced summary of what was done and what was found	2
**Introduction**			
Background/rationale	2	Explain the scientific background and rationale for the investigation being reported	4-5
Objectives	3	State specific objectives, including any prespecified hypotheses	5
**Methods**			
Study design	4	Present key elements of study design early in the paper	5-10
Setting	5	Describe the setting, locations, and relevant dates, including periods of recruitment, exposure, follow-up, and data collection	5-6
Participants	6	(*a*) Give the eligibility criteria, and the sources and methods of selection of participants. Describe methods of follow-up	5-10
		(*b*) For matched studies, give matching criteria and number of exposed and unexposed	n/a
Variables	7	Clearly define all outcomes, exposures, predictors, potential confounders, and effect modifiers. Give diagnostic criteria, if applicable	6-9 & Suppl methods
Data sources/ measurement	8*	For each variable of interest, give sources of data and details of methods of assessment (measurement). Describe comparability of assessment methods if there is more than one group	6-9
Bias	9	Describe any efforts to address potential sources of bias	9-10, 14
Study size	10	Explain how the study size was arrived at	9, Table S1
Quantitative variables	11	Explain how quantitative variables were handled in the analyses. If applicable, describe which groupings were chosen and why	6-9, Table 1, Table S2
Statistical methods	12	(*a*) Describe all statistical methods, including those used to control for confounding	9-10
		(*b*) Describe any methods used to examine subgroups and interactions	9-10
		(*c*) Explain how missing data were addressed	9-10, table S1
		(*d*) If applicable, explain how loss to follow-up was addressed	n/a

For peer review only

		(*e*) Describe any sensitivity analyses	10
**Results**			
Participants	13*	(a) Report numbers of individuals at each stage of study—eg numbers potentially eligible, examined for eligibility, confirmed eligible, included in the study, completing follow-up, and analysed	5-6, 9-10, footnotes to tables
		(b) Give reasons for non-participation at each stage	5-6
		(c) Consider use of a flow diagram	See Stafford et al 2013
Descriptive data	14*	(a) Give characteristics of study participants (eg demographic, clinical, social) and information on exposures and potential confounders	Table 1 and Table S2
		(b) Indicate number of participants with missing data for each variable of interest	Table S1 and Table S2
		(c) Summarise follow-up time (eg, average and total amount)	n/a
Outcome data	15*	Report numbers of outcome events or summary measures over time	Tables
Main results	16	(*a*) Give unadjusted estimates and, if applicable, confounder-adjusted estimates and their precision (eg, 95% confidence interval). Make clear which confounders were adjusted for and why they were included	10-11, Tables 2-3
		(*b*) Report category boundaries when continuous variables were categorized	Tables 1-3
		(*c*) If relevant, consider translating estimates of relative risk into absolute risk for a meaningful time period	-
Other analyses	17	Report other analyses done—eg analyses of subgroups and interactions, and sensitivity analyses	11-12
**Discussion**			
Key results	18	Summarise key results with reference to study objectives	12
**Limitations**			
Interpretation	20	Give a cautious overall interpretation of results considering objectives, limitations, multiplicity of analyses, results from similar studies, and other relevant evidence	12-15
Generalisability	21	Discuss the generalisability (external validity) of the study results	14
**Other information**			
Funding	22	Give the source of funding and the role of the funders for the present study and, if applicable, for the original study on which the present article is based	16

*Give information separately for cases and controls in case-control studies and, if applicable, for exposed and unexposed groups in cohort and cross-sectional studies.

For peer review only

Note: An Explanation and Elaboration article discusses each checklist item and gives methodological background and published examples of transparent reporting. The STROBE checklist is best used in conjunction with this article (freely available on the Web sites of PLoS Medicine at http://www.plosmedicine.org/, Annals of Internal Medicine at http://www.annals.org/, and Epidemiology at http://www.epidem.com/). Information on the STROBE Initiative is available at www.strobe-statement.org.

For peer review only
